# A new era in cancer therapy: targeting the Proteasome-Bcl-2 axis

**DOI:** 10.1186/s13046-025-03505-5

**Published:** 2025-08-21

**Authors:** Sourabh Soni, Vandana Anang, Yutong Zhao, Jeffrey C. Horowitz, Richard S. Nho, Yohannes A. Mebratu

**Affiliations:** 1 Department of Internal Medicine, Division of Pulmonary, Critical Care and Sleep Medicine, Davis Heart and Lung Institute, College of Medicine, The Ohio State University Wexner Medical Center, Columbus, OH USA; 2 Department of Physiology and Cell Biology, Davis Heart and Lung Institute, College of Medicine, The Ohio State University Wexner Medical Center, Columbus, OH USA

**Keywords:** Apoptosis, Bcl-2 family proteins, Cancer, Proteasome inhibitors, Therapeutic potential, Ubiquitination, Ubiquitin-proteasome system (UPS)

## Abstract

The B-cell lymphoma-2 (Bcl-2) family proteins, key regulators of apoptosis, are frequently dysregulated in cancer, tipping the balance of cell survival and apoptosis in favor of survival. The ubiquitin-proteasome system (UPS) is a critical cellular machinery that controls the Bcl-2 levels through regulation of protein stability. This review delves into the intricate interplay between the proteasome and Bcl-2 family members, exploring how proteasome-mediated degradation impacts cell survival and proliferation to influence cancer progression. We discuss the therapeutic potential of targeting the proteasome-Bcl-2 axis, including the use of proteasome inhibitors as anticancer agents. We examine their mechanisms of action, clinical efficacy, and limitations while exploring emerging strategies to overcome these challenges.

## Background

Programmed cell death, or apoptosis, is a tightly controlled process crucial for maintaining cellular homeostasis. This fundamental biological process plays a critical role in a myriad of physiological processes, including embryonic development [1], tissue remodeling [2], and immune response [3]. A delicate balance between cell survival and death is essential for proper tissue function. Imbalances in this equilibrium can contribute to a range of human diseases, such as cancer, neurodegenerative disorders, fibrotic and autoimmune diseases.

**Fig. 1:**
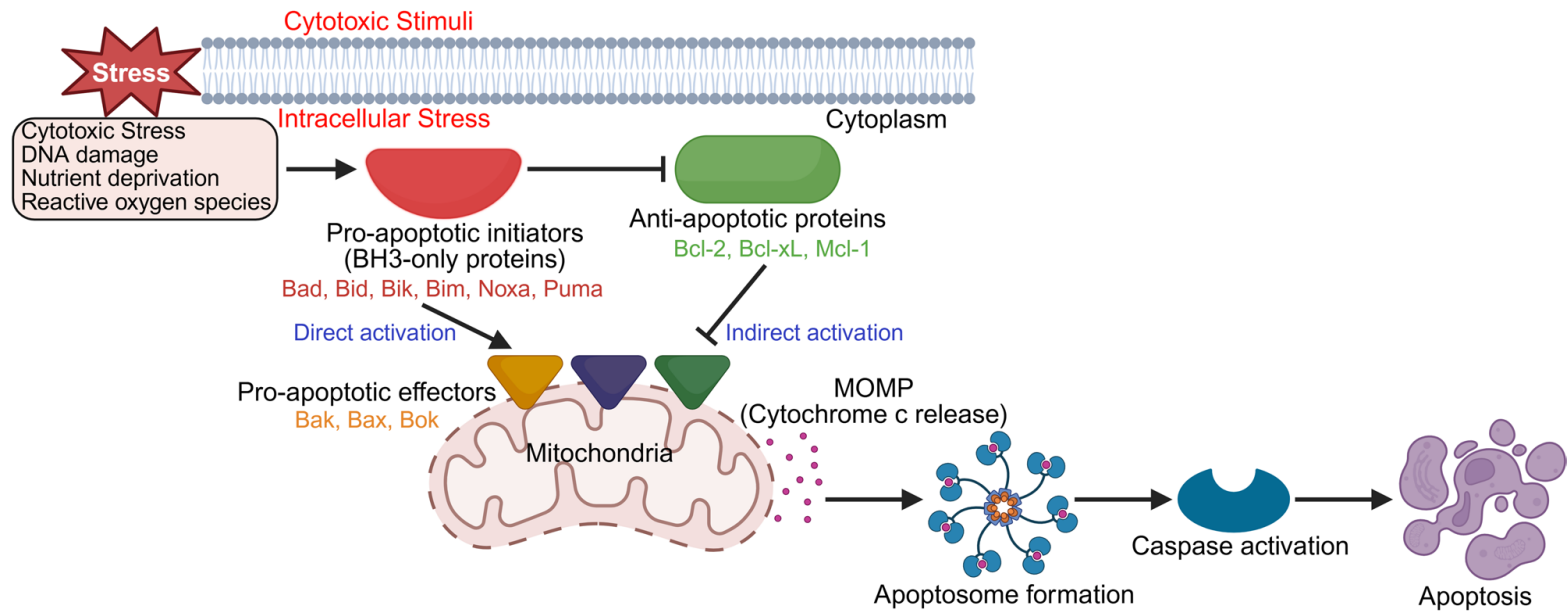
The intrinsic mitochondrial pathway of apoptosis: central role of Bcl-2 family proteins in stress-induced cell death. Cytotoxic stress signals such as DNA damage, nutrient deprivation, or reactive oxygen species initiate intracellular stress responses that activate BH3-only pro-apoptotic initiators (Bad, Bid, Bik, Bim, Noxa, Puma). These proteins either directly activate pro-apoptotic effectors (Bak, Bax, Bok) or indirectly promote their activation by neutralizing anti-apoptotic members (Bcl-2, Bcl-xL, Mcl-1). Once activated, Bak/Bax/Bok oligomerize at the mitochondrial outer membrane, causing mitochondrial outer membrane permeabilization (MOMP) and subsequent release of cytochrome c into the cytoplasm. This leads to apoptosome formation, caspase activation, and apoptosis. Arrows denote activation, while blunt-ended lines indicate inhibition. Red text indicates BH3-only pro-apoptotic initiators, orange indicates pro-apoptotic effectors, and green indicates anti-apoptotic proteins. Overall, this figure illustrates how Bcl-2 family members integrate cytotoxic stress signals to regulate mitochondrial-mediated apoptosis

Central to the regulation of apoptosis is the B-cell lymphoma-2 (Bcl-2) family of proteins [4]. This family comprises both anti-apoptotic (pro-survival) and pro-apoptotic members, which interact to maintain a delicate balance between cell survival and death (Table 1) [5]. Figure 1 illustrates how these proteins function downstream of cellular stress signals to govern mitochondrial outer membrane permeabilization and activation of the intrinsic apoptotic pathway. In cancer, this delicate balance is often skewed, allowing tumor cells to evade apoptosis and proliferate unchecked. This evasion frequently involves the dysregulation of Bcl-2 family proteins. Anti-apoptotic members are commonly overexpressed in cancer cells, actively inhibiting apoptosis and promoting tumor growth, while pro-apoptotic members may be downregulated or inactivated, further contributing to cancer cell survival and unchecked proliferation [6].

**Table 1:** Involvement of Bcl-2 family proteins in cancer

Bcl-2 Family Protein Classification	Protein	Associated Cancers	References
Anti-apoptotic proteins	Bcl-2	Breast	[26–31, 76, 202]
		Gastric	[25, 32, 33]
		Hematological	[34–40]
		Liver	[41–46]
		Lung	[47–51]
		Pancreas	[52–55]
		Prostate	[56, 57]
	Bcl-xL	Breast, Melanoma, Pancreas	[20, 58–61, 70]
	Mcl-1	Gynecologic, Lung, Melanoma,	[10, 20, 62–67, 70, 167]
Pro-apoptotic proteins (Effectors)	Bak	Breast, Colorectal, Gastric, Lung, Pancreas, Skin	[5, 15, 25, 69, 70, 187]
	Bax	Breast, Gastric, Liver	[15, 25, 42, 61, 70, 125, 149]
	Bok	Liver, Lung, Colorectal	[94–98]
Pro-apoptotic proteins (Initiators)	Bad	Ovary	[25, 68]
	Bid	Liver, Myeloma,	[71–74, 127]
	Bik	Breast	[75, 76, 79, 80, 83–86, 132]
		Colorectal	[50, 87]
		Gastric	[89]
		Melanoma	[82]
		Myeloma	[81]
		Prostate	[87, 88]
	Bim	Oral	[90–93]
	Noxa	Breast, Colorectal, Leukemia, Lung, Melenoma, Myeloma, Ovarian, Prostate, Rhabdosarcoma	[64–66, 99, 100, 167, 186]
	Puma	Breast, Colon, Prostate	[100–103, 149]

**Fig. 2:**
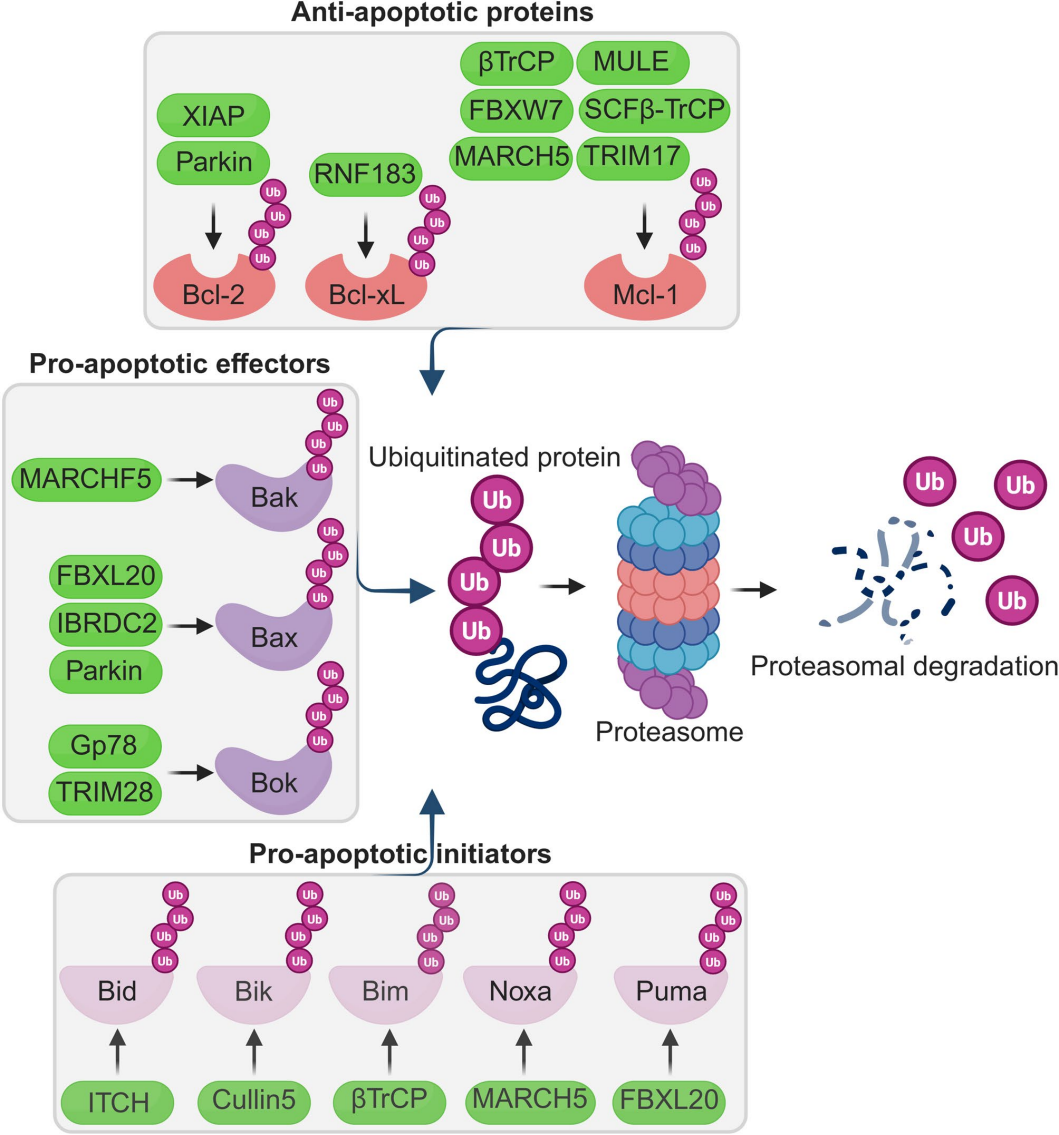
Ubiquitin-proteasome system (UPS)-mediated regulation of Bcl-2 family proteins. This figure illustrates the role of E3 ubiquitin ligases in targeting Bcl-2 family proteins for ubiquitination and subsequent proteasomal degradation. Central to the figure is the ubiquitin-proteasome system (UPS), where polyubiquitinated proteins are recognized and degraded by the 26 S proteasome. Proteins marked with multiple “Ub” (ubiquitin) icons are designated for degradation. **Top panel**: Anti-apoptotic Bcl-2 family proteins (Bcl-2, Bcl-xL, and Mcl-1) are regulated by specific E3 ligases (green ovals) which promote their ubiquitination. **Left panel**: Pro-apoptotic effector proteins (Bak, Bax, and Bok) are similarly targeted by specific E3 ligases (green ovals) to modulate apoptosis. **Bottom panel**: BH3-only pro-apoptotic initiator proteins (Bid, Bik, Bim, Noxa, Puma) are shown to be ubiquitinated by specific E3 ligases (green ovals). The central flow in the figure depicts the general UPS process, E3 ligases catalyze the attachment of ubiquitin to substrates, leading to recognition and degradation by the proteasome. Ubiquitinated proteins (tagged with purple Ub chains) enter the proteasome, are degraded, and free ubiquitin molecules are recycled. Green ovals indicate E3 ubiquitin ligases; arrows denote direction of ubiquitination or degradation. Shapes and colors of Bcl-2 family proteins represent their functional class: red (anti-apoptotic), purple (pro-apoptotic effectors), and pink (BH3-only proteins). Ub = Ubiquitin

The ubiquitin-proteasome system (UPS) is a vital cellular machinery that regulates protein stability by tagging proteins for degradation through ubiquitination. In the context of apoptosis, the UPS plays a pivotal role in determining the fate of pro- and anti-apoptotic Bcl-2 family members. As shown in Fig. 2, several E3 ubiquitin ligases specifically target different Bcl-2 family proteins for degradation, thereby fine-tuning apoptotic responses [7–9]. It functions by marking proteins for destruction by the 26 S proteasome, a multi-catalytic protease complex [10, 11]. Dysregulation of the UPS, particularly in cancer cells, can lead to the accumulation of anti-apoptotic proteins and the degradation of pro-apoptotic proteins, thereby amplifying cell survival and tumor growth [9, 12]. The specific levels of both pro- and anti-apoptotic Bcl-2 members, controlled by UPS activity, collectively determine a cell’s apoptotic threshold and its response to various apoptotic stimuli [13, 14]. This review delves into the intricate interplay between the proteasome and Bcl-2 family members, examining how proteasome-mediated degradation impacts cell survival, proliferation, and cancer progression. We will discuss the therapeutic potential of targeting the proteasome-Bcl-2 axis, particularly the use of proteasome inhibitors as anticancer agents. We will also explore their mechanisms of action, clinical efficacy, limitations, and emerging strategies to overcome these challenges.

## Overview of the Bcl-2 family members

The Bcl-2 family comprises a diverse group of proteins that critically regulate apoptosis, essentially acting as the gatekeepers of programmed cell death. This family is broadly categorized into two primary groups: pro-apoptotic and anti-apoptotic (pro-survival) proteins.

The pro-apoptotic proteins serve as the initiators and executioners of cell death. This group includes effector proteins like Bak, Bax, and Bok, as well as various Bcl-2 homology (BH) domain 3 (BH3)-only proteins (e.g., Bid, Bad, Bik, Bim, Puma, and Noxa) [15, 16]. In most healthy cells, Bax typically resides in the cytosol, while Bak is found at the mitochondrial outer membrane (MOM), both remaining dormant. Upon receiving pro-apoptotic signals, the BH3-only proteins act as sentinels of cellular stress. They trigger apoptosis either by directly activating Bax and Bak or by neutralizing the anti-apoptotic Bcl-2 proteins. Once activated, Bax and Bak undergo conformational changes, translocate to the MOM (if not already there), and oligomerize to form pores (Fig. 1). This crucial event, known as mitochondrial outer membrane permeabilization (MOMP), leads to the release of cytochrome c and other pro-apoptotic factors into the cytoplasm, thereby initiating the intrinsic apoptotic pathway [17, 18]. Later in this review (Section Bcl-2 family proteins in certain cancer types and Anti-apoptotic (pro-survival) Bcl-family proteins in cancer), we will delve into the specific mechanisms and regulatory insights concerning each of these critical pro-apoptotic Bcl-2 family members. Conversely, anti-apoptotic family members, such as Bcl-2, Bcl-xL, and Mcl-1 (detailed in Section Bcl-2 family proteins in certain cancer types and Anti-apoptotic (pro-survival) Bcl-family proteins in cancer), function as guardians of cell survival. These proteins typically possess multiple BH domains (BH1, BH2, BH3, BH4). Their primary mechanism of action involves binding to and sequestering pro-apoptotic proteins (especially Bax, Bak, and BH3-only proteins) (Fig. 1). By neutralizing these pro-death signals and directly blocking the formation of the mitochondrial permeability transition pore, anti-apoptotic proteins effectively prevent MOMP and the subsequent release of cytochrome c, thus maintaining cellular homeostasis and preventing unwanted cell death [19, 20].

## The Ubiquitin-Proteasome system (UPS)

UPS is essential for maintaining cellular homeostasis, directly impacting apoptosis and cancer cell survival by controlling the degradation and stabilization of intracellular proteins. The UPS operates through a cascade of enzymatic reactions involving ubiquitin-activating (E1), ubiquitin-conjugating (E2), and ubiquitin ligase (E3) enzymes. These enzymes tag proteins with ubiquitin molecules, marking them for degradation by the proteasome or for alternate cellular processes if proteasome-independent chains are employed [12]. This tagging system influences crucial cellular functions, including DNA replication, repair, cell cycle progression, and apoptotic signaling, all of which are highly relevant to cancer development and therapy resistance. E3 ligases play a pivotal role in UPS by identifying and binding specific target proteins (Fig. 2). There are three major classes of E3 ligases-RING, HECT, and RBR-each exerting unique influences on cellular function [12, 21, 22]. RING ligases mediate direct transfer of ubiquitin to targets, while HECT and RBR ligases act as intermediates.

Disruptions in UPS-mediated control of apoptotic proteins can lead to an imbalance favoring survival over cell death, a hallmark of cancer cells. Targeting specific E3 ligases or their substrates offers promising avenues for cancer therapy. For instance, proteasome inhibitors that block the degradation pathway can lead to the accumulation of pro-apoptotic factors, pushing cancer cells towards apoptosis. Moreover, selective targeting of anti-apoptotic proteins *via* E3 ligase modulators could sensitize cancer cells to chemotherapy. Similarly, modulating UPS components could enhance the efficacy of apoptosis-inducing treatments, potentially overcoming chemoresistance in tumors [23].

## Bcl-2 family proteins in certain cancer types

Due to their pivotal roles in governing cell survival and apoptosis, the delicate balance between pro- and anti-apoptotic Bcl-2 family proteins is critical for preventing cancer development and progression [4]. Overexpression of anti-apoptotic proteins (e.g., Bcl-2, Bcl-xL, Mcl-1) is a common hallmark across various cancers, often exhibiting distinct patterns of expression in different cancer types such as breast, gastric, lung, and prostate cancers [24] (Table 1). Understanding the precise mechanisms by which Bcl-2 family proteins drive cancer progression, drug resistance, and sustained cell survival is crucial for identifying effective therapeutic targets aimed at restoring apoptotic balance in tumor cells. The following sections will examine the specific contributions of individual Bcl-2 family proteins to tumorigenesis, metastasis, and therapeutic resistance.

## Anti-apoptotic (pro-survival) Bcl-family proteins in cancer

### B-cell lymphoma-2 (Bcl-2)

Elevated Bcl-2 protein levels are frequently observed across multiple cancer types, making Bcl-2 antagonists promising anti-cancer therapies [25]. This section critically examines Bcl-2’s specific involvement in various cancers, emphasizing its influence on tumor survival, therapeutic resistance, and its potential as a therapeutic target. While the ubiquitous role of Bcl-2 in promoting cell survival is well-established, its precise mechanisms and clinical implications can vary significantly across different cancer types, necessitating a nuanced understanding for effective therapeutic targeting.

#### Breast cancer

In breast cancer (BC) cells, the evasion of apoptosis is often facilitated by the upregulation of Bcl-2 expression [26], frequently driven by estrogen stimulation. Estrogen receptor (ERs) alpha (ERα) activation through recruitment of coactivators, directly induces Bcl-2 transcription by binding to the Bcl-2 promoter [27]. Clinically, this often translates to a more favorable initial response to endocrine therapy in ERs-positive (ER+) cases, suggesting that Bcl-2 acts as a key survival factor in these contexts, making cells dependent on hormonal pathways [28].

However, the relationship between Bcl-2 and therapy resistance in BC is complex and often paradoxical. While high Bcl-2 expression is initially associated with favorable prognostic characteristics such as lower histological grade, lower proliferation index, and positive hormone receptor status, this correlation often diminishes or is lost after progression on hormonal therapy [29, 30]. This suggests that as tumors develop resistance to endocrine therapy, they may shift away from ERs-mediated Bcl-2 upregulation, potentially relying on alternative survival pathways or constitutive Bcl-2 expression to maintain viability despite reduced ERs activity. Furthermore, despite its association with otherwise favorable characteristics, high Bcl-2 expression can also be correlated with an increased risk of lymph node metastasis [31]. This dichotomy highlights a critical gap in our understanding, while Bcl-2 may initially predict a better response to endocrine therapy in ER + tumors, its persistent high expression can also contribute to metastatic potential or drug resistance mechanisms independent of ERs signaling. Future research should focus on deciphering these context-dependent roles of Bcl-2 to inform more precise therapeutic strategies.

#### Gastric cancer

Bcl-2’s overexpression in gastric cancer (GC) is consistently linked to high malignancy and poor patient survival. A key regulatory mechanism involvesmiR-383, a microRNA that negatively regulates Bcl-2 by targeting its 3′-untranslated region to inhibit translation. Lower levels of miR-383 correlate with increased Bcl-2 expression in GC, underscoring Bcl-2’s pivotal role in GC pathogenesis [32]. This inverse relationship is particularly relevant as lower miR-383 levels are also associated with tumor cell resistance to 5-fluorouracil (5-FU), suggesting that elevated Bcl-2 contributes to drug resistance by inhibiting chemotherapy-induced apoptosis.

Beyond miR-383, Bcl-2’s contribution to drug resistance in GC is multifaceted. Its elevated expression, even in early-stage disease [25], promotes tumor growth and survival by maintaining anti-apoptotic signaling, allowing cancer cells to evade standard chemotherapy-induced cell death. More recent studies highlight Bcl-2’s involvement in promoting GC metastasis by enhancing cancer stemness and resistance to targeted therapies. Specifically, Bcl-2 can foster cancer stemness by preventing apoptosis in the cancer stem cell population, allowing these highly tumorigenic and drug-resistant cells to survive and proliferate. It also contributes to resistance against targeted therapies by providing an alternative pro-survival pathway when other oncogenic signals are inhibited [33]. These findings strongly suggest that targeting Bcl-2, perhaps through small-molecule inhibitors or gene silencing, offers a promising personalized therapeutic approach for aggressive GC, potentially overcoming drug resistance and suppressing metastasis by re-sensitizing cancer cells to apoptotic stimuli and reducing the survival advantage of stem-like cells. Given that GC is often diagnosed in advanced stages, the evidence of Bcl-2’s early-stage involvement and its role in drug resistance provides a strong rationale for exploring Bcl-2 inhibitors, perhaps in combination therapies, to improve treatment outcomes at all stages of disease.

#### Hematological malignancies

Bcl-2 plays a pivotal role in hematological malignancies with its overexpression driving leukemic cell survival, a key mechanism in the pathogenesis of diseases like chronic lymphocytic leukemia (CLL). The development of venetoclax, a selective Bcl-2 inhibitor, has revolutionized CLL treatment by effectively inducing apoptosis in Bcl-2-dependent CLL cells. Initial clinical trials in relapsed/refractory (R/R) CLL and small lymphocytic lymphoma demonstrated a remarkable 79% overall response rate (ORR), with remarkable 15-month progression-free survival rate of 69%, significantly surpassing historical outcomes with conventional chemotherapy [34]. Subsequent studies confirmed its efficacy, even in high-risk subsets such as 17p-deleted R/R CLL, achieving a similar 79% ORR [35].

However, venetoclax monotherapy often faces limitations due to drug toxicity and compliance challenges [36, 37]. This has led to the exploration of combination strategies, with the triple combination of venetoclax with anti-CD20 monoclonal antibodies and BTK inhibitors showing promising deep and durable responses with manageable safety profiles [38].

Bcl-2 is also a critical player in acute myeloid leukemia (AML), promoting leukemic cell survival and contributing to resistance against conventional therapies by upregulating anti-apoptotic proteins. While venetoclax monotherapy demonstrated limited clinical activity (19% ORR) in patients unfit for intensive chemotherapy [39], its true therapeutic potential in AML lies in combination strategies. For example, combining venetoclax with hypomethylating agents like azacitidine and decitabine has significantly enhanced remission rates and prolonged survival in elderly AML patients. The landmark VIALE-A trial showed that venetoclax plus azacitidine nearly doubled overall survival compared to azacitidine alone (14.7 vs. 6 months) and led to a substantially higher complete remission (CR) rate [40]. These findings unequivocally highlight the critical role of Bcl-2 inhibition in AML therapy and underscore the ongoing need to refine venetoclax-based treatment strategies for improved patient outcomes. Future research should explore optimal combination regimens and identify biomarkers to predict response and mitigate resistance.

#### Hepatocellular carcinoma

Bcl-2 expression is significantly elevated in hepatocellular carcinoma (HCC) tissue at both the mRNA and protein levels, and this overexpression is strongly associated with increased tumor cell [41, 42]. The anti-apoptotic function of Bcl-2 allows HCC cells to evade the cell death induced by conventional chemotherapy, contributing to treatment failure. For instance, studies have shown that gansu ammonia goose deoxycholic acid sodium glycochenodeoxycholate, a bile acid derivative found in some traditional Chinese medicines, can paradoxically promote resistance in HCC cells by upregulating Bcl-2 expression and enhancing its phosphorylation [43]. This highlights how Bcl-2 can be modulated by various factors to further contribute to drug resistance in HCC, posing a challenge for therapeutic intervention.

The pivotal role of Bcl-2 in mediating chemoresistance in HCC positions it as a promising therapeutic target. While selective Bcl-2 inhibitors like venetoclax have demonstrated remarkable success in hematological malignancies, their application in solid tumors like HCC is still under investigation. Preclinical studies are exploring the efficacy of these inhibitors, either as single agents or in combination with existing chemotherapies or targeted therapies, to overcome Bcl-2-mediated resistance in HCC [44–46]. These ongoing efforts underscore the critical need to develop effective Bcl-2-targeted strategies to improve outcomes for HCC patients, particularly given the challenges of drug resistance in this aggressive cancer. Further research is needed to translate these preclinical findings into successful clinical applications.

#### Lung cancer

Abnormal Bcl-2 expression plays a significant role in lung cancer development and progression by preventing lung cancer cells with irreparable genetic changes from undergoing apoptosis, thereby contributing to their survival and proliferation and potentially leading to tumorigenesis [47]. However, Bcl-2’s role as a biomarker in lung cancer is more complex and context-dependent than in some other malignancies. While Bcl-2 generally promotes cell survival and can contribute to chemoresistance, some studies in non-small cell lung cancer (NSCLC) have shown a paradoxical association with favorable outcomes. For example, elevated Bcl-2 expression has been observed to identify a subgroup of NSCLC patients with improved overall survival (OS) and disease-specific survival (DSS) [48, 49]. Similarly, in lung squamous cell carcinoma, a subtype of NSCLC, elevated Bcl-2 expression is associated with a favorable prognosis, suggesting its potential as a prognostic biomarker for better survival [50].

The exact mechanisms underlying this paradoxical association remain largely unclear and warrant further investigation. Possible explanations include the specific cellular context, the co-expression of other pro-apoptotic or anti-apoptotic proteins, or the interaction of Bcl-2 with specific signaling pathways unique to certain lung cancer subtypes.

It is important to clarify that serum Bcl-2 refers to the presence of Bcl-2 protein in the bloodstream, often released from dying or damaged cells, including tumor cells. While the direct mechanism by which serum Bcl-2 impacts lung cancer cell apoptosis is not fully elucidated, elevated levels of circulating Bcl-2 could potentially reflect a higher tumor burden or a greater overall anti-apoptotic activity within the tumor, which might indirectly correlate with disease progression or response to therapy. However, the primary focus in lung cancer research, as in other cancers, is usually on the intracellular expression of Bcl-2 within the tumor cells themselves, as this directly influences their apoptotic machinery. Further research is needed to fully understand the clinical implications and utility of serum Bcl-2 levels in lung cancer patients [51]. Deciphering the precise contextual roles of Bcl-2 in lung cancer is crucial for its potential as a therapeutic target or prognostic biomarker.

#### Pancreatic cancer

Pancreatic cancer is characterized by a particularly poor clinical prognosis, with intrinsic and acquired resistance to chemotherapy, especially gemcitabine, posing a major therapeutic challenge. Bcl-2 inhibition has emerged as a promising strategy to overcome this resistance [52]. A significant mechanism of gemcitabine resistance in pancreatic cancer cells is the paradoxical induction of Bcl-2 overexpression by chemotherapy itself. This upregulation allows cancer cells to evade the pro-apoptotic effects of the drug. Venetoclax, a selective Bcl-2 inhibitor, effectively counteracts this by downregulating gemcitabine-induced Bcl-2 overexpression, thereby promoting apoptosis and significantly increasing the sensitivity of pancreatic cancer cells to gemcitabine [53]. This mechanism, where chemotherapeutic agents can induce pro-survival proteins like Bcl-2, is a recognized form of acquired drug resistance observed in various cancer types [54].

A significant concern with earlier generations of Bcl-2 inhibitors was their off-target effects, specifically the induction of sustained Ca²⁺ responses in normal pancreatic acinar cells. These sustained Ca²⁺ responses refer to a prolonged and uncontrolled elevation of intracellular calcium levels, which can lead to cellular dysfunction, toxicity, and even cell death in healthy tissues. This was a particular concern for the pancreas due to its sensitivity to calcium dysregulation. However, venetoclax demonstrates an improved safety profile by preserving intracellular calcium homeostasis in normal cells [55]. This selectivity of venetoclax for Bcl-2, without broadly disrupting calcium signaling in healthy pancreatic tissue, is crucial for its potential clinical application in pancreatic cancer. Given the challenges of treating pancreatic cancer, the selective inhibition of Bcl-2 by venetoclax offers a promising avenue to enhance the efficacy of existing chemotherapies while minimizing systemic toxicity.

#### Prostate cancer

Overexpression of Bcl-2 is a critical factor in prostate cancer progression, facilitating tumor growth and increasing tumor cell survival [25]. This anti-apoptotic effect becomes particularly relevant in advanced stages of the disease. Recent studies have highlighted that Bcl-2 is significantly upregulated in androgen receptor-independent, neuroendocrine-like castration-resistant prostate cancer (CRPC). This overexpression contributes to resistance against androgen receptor signaling inhibitors (ARSI), which are standard treatments for prostate cancer, and is associated with shorter OS in patients [56].

The regulation of Bcl-2 expression in prostate cancer is complex and involves mechanisms such as DNA methylation and the influence of lineage plasticity factors like ASCL1 (Achaete-scute homolog 1). For example, hypomethylation in the Bcl-2 promoter region can lead to increased gene expression, while factors driving neuroendocrine differentiation, such as ASCL1, can also contribute to Bcl-2 upregulation, fostering a more aggressive, treatment-resistant phenotype [56]. While this section is the first instance of discussing DNA methylation and ASCL1 in relation to Bcl-2 regulation, it’s important to note that epigenetic mechanisms (like DNA methylation) and transcription factors influencing cell plasticity are broad regulatory principles that can affect gene expressions, including Bcl-2, across various cancer types. Their specific involvement and prominence in driving Bcl-2 expression can vary depending on the cancer type and its molecular subtypes. This detailed discussion is presented here as it is particularly relevant to the context of ARSI resistance and neuroendocrine differentiation in prostate cancer.

Given its crucial role in promoting resistance and progression, Bcl-2 presents a promising therapeutic target in prostate cancer. However, monotherapy with Bcl-2 inhibitors may not be sufficient, and combination strategies are often necessary to enhance tumor cell apoptosis and improve treatment response [56]. Furthermore, Bcl-2 plays a critical role in promoting the transition from androgen-dependent to androgen-independent growth, a critical step in prostate cancer evolution, with its expression significantly upregulated in androgen-independent prostate cancer cells [57]. Preclinical models have shown that silencing Bcl-2 enhances apoptosis and suppress tumor growth, further underscoring its potential as a therapeutic target in advanced prostate cancer [57]. Future research should focus on developing effective combination therapies that leverage Bcl-2 inhibition to overcome ARSI resistance and improve outcomes for patients with advanced prostate cancer.

### B-cell lymphoma-extra large (Bcl-xL)

Bcl-xL is a critical anti-apoptotic protein and a key regulator of tumor survival, progression, and chemoresistance. Its overexpression contributes to various cancer hallmarks, including tumor cell growth, metastasis, and maintenance of the cancer stem cell phenotype [58]. Bcl-xL protein expression levels are significantly elevated in many cancer cells compared to normal cells. Its primary anti-apoptotic mechanism involves binding to and inhibiting pro-apoptotic Bcl-2 family members like Bax and Bak, thereby preventing the MOMP and subsequent release of cytochrome c, which is essential for initiating the apoptotic cascade [59].

Beyond its canonical apoptosis inhibition, a notable and increasingly recognized role of Bcl-xL lies in its ability to actively promote epithelial-mesenchymal transition (EMT), cellular migration, invasion, and stemness in various cancer cell lines, including pancreatic neuroendocrine and BC cell lines [59]. Crucially, these functions often appear to be independent of its anti-apoptotic activity, suggesting that Bcl-xL can influence tumor aggressiveness through diverse cellular processes beyond simply preventing cell death. This multifaceted role differentiates Bcl-xL from other anti-apoptotic proteins like Bcl-2, whose direct involvement in EMT is less clearly established. Moreover, Bcl-xL contributes significantly to chemoresistance through complex interactions with oncogenic signaling pathways, such as the RAS pathway, and its influence on EMT and cancer-initiating cells [60]. Hyperactive RAS signaling, common in many cancers, can activate downstream survival pathways, and Bcl-xL can bolster these pro-survival signals, thereby making cancer cells more resistant to chemotherapy. This direct interplay with oncogenic pathways highlights a distinct mechanism of resistance compared to Bcl-2’s primary role, which is often directly tied to preventing apoptosis by sequestering pro-apoptotic proteins.

These multifaceted findings underscore Bcl-xL as a key driver of cancer progression and chemoresistance. However, targeting Bcl-xL alone is often insufficient to induce robust apoptosis in many cancer cells [61]. This mechanistic difference from Bcl-2 can be attributed to several factors. Cells may exhibit a higher dependency on other anti-apoptotic Bcl-2 family members (e.g., Mcl-1 or Bcl-2 itself) to survive, or they might not be primed for apoptosis, meaning they have insufficient levels of pro-apoptotic proteins or high levels of other survival factors that render them less sensitive to single-agent Bcl-xL inhibition. Functional assays like BH3 profiling, which assess the apoptotic priming of cells, consistently demonstrate that cells resistant to Bcl-xL inhibitors often rely on other anti-apoptotic proteins and/or are not sufficiently primed for apoptosis, thus requiring broader inhibition of anti-apoptotic pathways to achieve cell death [61]. Nevertheless, precisely because of its diverse roles beyond apoptosis inhibition, targeting Bcl-xL, particularly in combination with other therapeutic agents, represents a promising strategy to overcome drug resistance and improve cancer treatment outcomes. Future research should focus on identifying the specific contexts where Bcl-xL’s non-apoptotic functions are dominant and developing combination therapies that effectively target both its anti-apoptotic and pro-metastatic roles.

### Myeloid cell leukemia-1 (Mcl-1)

Mcl-1 is a crucial anti-apoptotic protein, frequently overexpressed in various cancers, including NSCLC, where it significantly contributes to chemoresistance [62]. Like other anti-apoptotic members of the Bcl-2 family (e.g., Bcl-2 and Bcl-xL), Mcl-1’s primary function is to maintain cell survival by preventing the activation of the intrinsic apoptotic pathway.

Mcl-1’s expression is tightly regulated at both the transcriptional and post-transcriptional levels. A key pathway influencing its expression is the PI3K/AKT signaling pathway, which is often hyperactive in cancer cells. This pathway increases Mcl-1 expression during processes like myeloid differentiation and in response to cytokine stimulation, thereby promoting cell survival [63].

One of the central mechanisms by which Mcl-1 confers resistance to apoptosis is by sequestering pro-apoptotic Bcl-2 family members, particularly Bak (and to a lesser extent, Bax). In healthy cells, Bak remains in an inactive state, but upon an apoptotic stimulus, it undergoes conformational changes and oligomerization to form pores in the mitochondrial outer membrane, leading to the release of pro-apoptotic factors like cytochrome c. Mcl-1 binds to Bak, preventing its activation and subsequent MOMP, thereby blocking the intrinsic apoptotic cascade. This sequestration by Mcl-1 is a major cause of resistance to BH3 mimetics like ABT-737, which are designed to mimic pro-apoptotic BH3-only proteins and induce cell death by displacing anti-apoptotic proteins [64].

To counteract Mcl-1’s protective effects, several strategies are being developed. For instance, increasing the expression of Noxa, another BH3-only protein, can overcome Mcl-1-mediated resistance. Noxa preferentially binds to Mcl-1 with high affinity, disrupting the Mcl-1/Bak complex. This frees Bak, allowing it to activate and initiate apoptosis, thereby potentially enhancing the therapeutic efficacy of treatments like ABT-737 [64]. This highlights the delicate balance of interactions within the Bcl-2 family, where the relative expression and binding affinities of pro- and anti-apoptotic members dictate a cell’s susceptibility to apoptosis. The high affinity of Noxa for Mcl-1, in contrast to its weaker binding to Bcl-2 or Bcl-xL, positions it as a specific tool to target Mcl-1-dependent survival.

The stability and degradation of Mcl-1 are also critical regulatory points that can be therapeutically exploited. Mcl-1’s stability is influenced by the mitochondrial E3 ubiquitin ligase MARCH5, which can ubiquitinate Mcl-1, marking it for degradation. Furthermore, the intricate Noxa/Mcl-1 axis plays a role, as Noxa binding can influence Mcl-1’s susceptibility to degradation [65]. Targeting MARCH5 or modulating the activity of proteins like Noxa and Usp9x (ubiquitin-specific protease 9, an enzyme that deubiquitinates and thereby stabilizes Mcl-1) can promote Mcl-1 degradation. This reduction in Mcl-1 levels enhances apoptosis and diminishes resistance to various therapeutic agents, including ABT-737 [66]. Therapeutic strategies such as the use of pemetrexed, an antifolate chemotherapy drug, have shown promise in preclinical and clinical settings by disrupting the Noxa-Usp9x-Mcl-1 pathway, highlighting the significant potential of Mcl-1-targeted interventions to improve cancer treatment outcomes [62]. Given Mcl-1’s frequent overexpression and its role in chemoresistance, particularly in cancers reliant on Mcl-1 for survival, selective inhibitors targeting Mcl-1 are actively being developed [67]. These inhibitors aim to bypass the endogenous UPS-mediated stabilization mechanisms, thereby promoting Mcl-1 degradation and enhancing apoptosis. Future research needs to refine these targeted approaches and address potential compensatory mechanisms that may arise during Mcl-1 inhibition.

## Pro-apoptotic Bcl-family proteins

### Bcl-2-associated agonist of cell death (Bad)

Bad’s pro-apoptotic effect is mediated by forming heterodimers with Bcl-2, inhibiting Bcl-2’s anti-apoptotic function. High Bad expression is strongly associated with tumor cell apoptosis, making Bad enhancement an attractive strategy for cancer therapy [25]. However, the cellular context significantly impacts Bad’s activity. In epithelial ovarian cancer, for instance, activated AKT phosphorylates Bad at Ser136 which effectively disables Bad’s crucial role in initiating cell death, making these cancer cells less responsive to cisplatin chemotherapy [68]. This phosphorylation represents a critical regulatory point where oncogenic signaling pathways can directly antagonize pro-apoptotic proteins. Researchers are actively investigating the precise levels of both active and phosphorylated Bad (pBad at Ser136), comparing these levels before and after AKT inhibition, and observing how cisplatin affects these dynamics. Understanding this phosphorylation of Bad is critical, as it directly contributes to tumor cell survival and can undermine the effectiveness of pro-apoptotic proteins, driving cancer’s resistance to treatment [68]. A key knowledge gap lies in fully characterizing the kinases and phosphatases that regulate Bad phosphorylation in various cancer types, and how this regulation might be exploited therapeutically to restore Bad’s pro-apoptotic function.

### Bcl-2 homologous antagonist killer (Bak)

Bak is a significant apoptotic effector protein located on the mitochondrial outer membrane, acting as a crucial instigator of programmed cell death when triggered by apoptotic signals. Bak shares significant structural similarities with Bcl-2, particularly in its BH domains. While Bcl-2 is known for preventing cell death, this structural similarity is vital because it allows Bak to directly compete with or bind to the anti-apoptotic Bcl-2 proteins, disrupting their ability to protect the cell [5]. By doing so, Bak effectively shifts the balance towards cell death.

The clinical relevance of Bak expression in cancer, however, presents a complex and sometimes contradictory picture. Previous research has associated a lack of Bak expression with various cancers, including colorectal, gastric, pancreatic, and skin cancers, suggesting a tumor suppressive role for Bak in these contexts [25]. This aligns with the expectation that functional pro-apoptotic proteins would be downregulated in cancer. In contrast, high levels of Bak in breast cancer have been linked to better patient prognosis [69]. This apparent contradiction highlights a key area of disagreement in the literature and suggests that Bak’s role is highly context-dependent, potentially influenced by co-expression of other Bcl-2 family members, specific genetic mutations, or post-translational modifications within the tumor. Future research needs to systematically investigate these contextual factors to fully elucidate Bak’s prognostic and predictive value across different cancer types.

### Bcl-2-associated X protein (Bax)

The complex regulation of apoptosis by Bax, along with its intricate interplay with anti-apoptotic proteins such as Bcl-xL and Bcl-2, plays a key role in influencing cancer cell survival and resistance to treatment [25]. Bax is a critical executioner of the intrinsic apoptotic pathway. Upon receiving apoptotic signals, Bax undergoes conformational changes, translocates to the mitochondrial outer membrane, and oligomerizes, leading to the formation of pores that release pro-apoptotic factors into the cytoplasm.

Anti-apoptotic proteins tightly regulate Bax activation. Bcl-xL plays a crucial role in regulating Bax activation, particularly in solid tumor cell lines, where Bcl-xL and Bcl-2 exhibit stronger binding to Bax compared to Mcl-1 [61, 70]. This means that in many solid tumors, even if Mcl-1 is present, the primary anti-apoptotic pressure against Bax comes from Bcl-xL and Bcl-2. In the absence of Bcl-2 expression, Bcl-xL often becomes the predominant anti-apoptotic protein that sequesters activated Bax in solid tumors [61, 70]. This highlights a key difference in the relative importance of anti-apoptotic proteins in different tumor types. In contrast, in hematological malignancies, where both Bcl-xL and Bcl-2 are typically expressed at similar levels, both proteins contribute significantly to influencing Bax activation and thereby cell fate. The efficacy of Navitoclax, a dual Bcl-2/Bcl-xL inhibitor, is largely determined by the levels of available, active Bax, as its ability to induce apoptosis relies on the subsequent activation of Bax [61].

In HCC, Bcl-2 can hinder apoptosis by not only forming inhibitory complexes with Bax but also blocking the Fas/FasL apoptosis pathway [25]. The Fas/FasL pathway is an extrinsic apoptotic pathway, where Fas (a death receptor on the cell surface) binds to its ligand, FasL, leading to the activation of caspase cascades and cell death. Bcl-2 can interfere with this pathway at various points, thereby contributing to resistance. Bcl-2’s overexpression and phosphorylation play a vital role in regulating cell proliferation, contributing significantly to tumor growth and multidrug resistance in HCC [25, 42]. The central role of Bax as an executioner protein makes its activation a critical bottleneck for therapeutic strategies aimed at inducing apoptosis. Understanding the precise mechanisms by which anti-apoptotic proteins sequester Bax in different cancer contexts remains a key area for further investigation.

### BH3-interacting domain death agonist (Bid)

Bid and its cleaved form, tBid, are often overexpressed in chemoresistant cancer cells, placing them in a primed apoptotic state. However, interactions with anti-apoptotic proteins like Bcl-2 can sequester tBid and block apoptosis [71]. Targeting these interactions with small molecule inhibitors may release tBid and selectively trigger cell death in resistant tumors. Recent studies have shown that high expression of the pro-apoptotic protein Bid sensitizes tumor cells to spindle assembly checkpoint (SAC) inhibitors making Bid essential for SAC abrogation-induced apoptosis [72]. This highlights a specific functional role for Bid in overcoming certain types of drug resistance.

Bid is expressed in liver metastases, where its levels are often higher than in non-tumorous liver tissue [73]. These findings suggest that Bid could be a potential biomarker to predict responsiveness to SAC-targeting therapies in solid tumors. Impaired death receptor signaling, involving the Fas-associated protein with death domain/Bid axis, has been shown to drive resistance to T-cell mediated immunotherapies in multiple myeloma [74]. This is a crucial insight as it extends Bid’s relevance beyond conventional chemotherapy. While conventional therapies may sometimes remain effective even with altered Bid signaling, Bid-dependent apoptotic signaling appears to be crucial for responses to novel immunotherapies, highlighting Bid as a key determinant of immunotherapy sensitivity in multiple myeloma. This emerging role of Bid in immunotherapy response represents a significant gap in our understanding and a promising avenue for future research.

### Bcl-2-interacting killer (Bik): dual roles in apoptosis

Bik is implicated in the regulation of tumor growth across various cancer types. In BC, the association of Bik expression with pathological complete response has been observed, even when analyzing patients receiving standard preoperative chemotherapy [75]. This aligns with numerous reports suggesting a pro-apoptotic, tumor suppressor function of Bik in specific tissues [76] and some studies have linked the absence of Bik expression with resistance to chemotherapeutic agents [77]. Furthermore, high levels of p-Erk1/2 correlate with decreased survival in BC patients, suggesting that Erk1/2-mediated Bik degradation plays a role in chemotherapy resistance, such as to 5-fluorouracil, and impacts disease progression [78]. This provides a mechanistic link for Bik’s potential positive prognostic value.

However, paradoxical findings exist. It was reported that Bik overexpression was associated with lower OS and shorter disease-free survival in breast cancer [79]. This significant discrepancy may be attributed to variables such as clinical stage, patient age, and menopausal status. For instance, studies in BC cell lines demonstrate that Bik’s subcellular localization differs across stages: in MCF-7 cells (pre-metastatic), Bik is cytosolic and translocates to the nucleus on cisplatin exposure; in MDA-MB-231 cells (metastatic), Bik is nuclear and remains so despite oxidative stress; and in MCF10 cells (normal), Bik remains cytosolic [80]. Thus, in locally advanced breast cancer, pre-treatment Bik localization could profoundly influence its function and clinical impact. This highlights a critical gap in our understanding of how Bik’s localization and context-dependent interactions dictate its pro- or anti-tumorigenic roles.

Beyond breast cancer, ectopic expression of Bik induces apoptosis in myeloma, colon, prostate, and melanoma cells [81, 82]. In MCF-7/BUS cells, Bik is induced by anti-estrogens, playing a critical role in antiestrogen-provoked apoptosis [83]. Yet, the association of Bik expression with poorer prognosis in BC [79] suggests it may induce inefficient or incomplete apoptosis in clinical settings, or that its expression is a marker of other underlying aggressive tumor features.

Mechanistically, Bik activates caspases and induces genomic damage through caspase-activated DNase. Its induction in ER + cells in response to estrogen signaling blockade suggests its relevance in ER + BC [83, 84]. In line with this, Bik elevation in ER + BC patients is associated with increased recurrence and mortality [85]. A direct correlation between Bik mRNA and ERα expression in BC tissue further supports ERα-mediated apoptosis pathway [86].

In vitro studies in PC-3 and HT-29 cells show Bik induces apoptosis by releasing cytochrome c, activating caspases, and causing DNA fragmentation [87]. Deletion of the Bik BH3 domain abolishes these effects. Intratumoral injection of a Bik-expressing adenovirus, but not the BH3-deleted version, suppresses PC-3 and HT-29 xenograft growth, with histological evidence of apoptosis. Furthermore, microRNA 486-3p directly targets Bik in colorectal cancer, regulating apoptosis and invasion [50]. In prostate cancer, KLF4 upregulates Bik expression, promoting cisplatin-induced apoptosis [88]. In GC, MyoD1 directly regulates Bik, inducing apoptosis [89]. Given these contradictory clinical findings and varied mechanistic roles, future research must systematically characterize Bik’s functions across different cancer types and cellular contexts, focusing on its post-translational modifications and subcellular localization, to resolve these discrepancies and fully exploit its therapeutic potential.

### Bcl-2 interacting mediator of cell death (Bim): linking apoptosis to tumor development

Bim, a pro-apoptotic protein, is typically maintained in an inactive state through association with microtubules or complexes with pro-survival proteins [90]. Bim is widely expressed in a variety of normal tissues, including epithelial, reproductive, hematopoietic, and nerve cells [91]. Bim induces apoptosis only after dissociating from its cytoplasmic protein complex following various cellular stimulation, such as DNA damage, growth factor withdrawal, or cytotoxic stress [92]. Once dissociated, Bim can then directly activate pro-apoptotic Bax and Bak, leading to their conformational change and oligomerization at the mitochondrial outer membrane. This initiates MOMP and the subsequent release of pro-apoptotic factors like cytochrome c, which triggers the caspase cascade and culminates in controlled cell death (apoptosis).

Increased Bim gene expression has been shown to effectively elevate apoptosis rates in tumor cells, making it a desirable target for cancer therapy. Recent research has correlated low Bim expression with more aggressive tumor phenotypes, such as squamous cell carcinoma histology, increased tumor aggressiveness, and enhanced proliferation [93]. This inverse relationship between Bim expression and tumor progression highlights Bim’s critical role as a tumor suppressor and its potential as a prognostic marker and therapeutic target in cancer. While the direct pro-apoptotic role of Bim is well-established, a significant gap in knowledge lies in fully understanding the precise upstream signaling pathways and post-translational modifications that regulate its release from inhibitory complexes in various cancer types, which could inform strategies to unleash its tumor-suppressive activity.

### Bcl-2-related ovarian killer (Bok): A novel regulator of tumor growth and survival

Recent research has highlighted the involvement of Bok, a lesser-known member of the Bcl-2 family, in promoting lung cancer progression. Studies using Bok-deficient mice expressing mutant Kras (KrasG12D) revealed a significant reduction in tumor burden, evidenced by decreased lesion number and histological grade [94]. These findings suggest that Bok promotes Kras-driven lung cancer progression in a p53-dependent manner, highlighting its potential as a therapeutic target [94]. This p53-dependency is a critical insight, indicating that Bok’s pro-tumorigenic activity might be context-specific and rely on the status of other tumor suppressor pathways.

In Bok knockout mice, Bok deficiency resulted in fewer and lower-grade tumors, primarily due to decreased cell proliferation. Crucially, these effects were abolished in the absence of functional p53, indicating Bok’s tumor-promoting role is indeed dependent on p53. In colorectal carcinoma cells, reducing Bok levels led to cell cycle arrest through increased p19INK4d, p21cip1, and p53 upregulation, with p53 absence nullifying Bok-driven effects [95, 96]. Similarly, in a mouse hepatocellular carcinoma model, Bok deficiency protected against diethylnitrosamine-induced liver apoptosis, resulting in fewer and smaller tumors [95]. Bok-deficient cells exhibited slower proliferation, supporting growth defects observed in various model [96, 97].

The clinical prognostic implications of Bok expression are, however, still emerging and show some complexity. In colorectal cancer, lower Bok protein levels correlate with later-stage tumors and better outcomes, suggesting a potential pro-apoptotic or tumor-suppressive role in this context [98]. Conversely, elevated Bok levels in tumors are linked to early recurrence and poor survival [98]. This apparent contradiction regarding its prognostic value underscores the need for more extensive and consistent clinical studies. The lack of correlation between elevated Bok levels and endoplasmic reticulum (ER) stress markers [98] further suggests that Bok’s diverse activities may impact cancer progression and prognosis through mechanisms beyond simple ER stress response. A significant gap remains in fully understanding the precise mechanisms by which Bok influences tumor proliferation and how its expression correlates with clinical outcomes across a broader range of cancer types, moving beyond mouse models to human cohorts.

### Phorbol-12-myristate-13-acetate-induced protein 1 (PMAIP1 commonly known as Noxa): A Key Prognostic Marker in Oncogenesis

Noxa, a BH3-only protein, is a critical pro-apoptotic effector involved in the pathogenesis of various cancers, including lung cancer, leukemias, rhabdomyosarcoma, prostate cancer, ovarian cancer, colorectal cancer, melanoma, and multiple myeloma [66]. Its selective high-affinity binding to Mcl-1 distinguishes it from other BH3-only proteins, making Noxa-specific drugs or BH3 mimetics targeting Mcl-1 a promising therapeutic strategy.

Noxa expression can be tightly regulated under specific conditions. For example, KRAS G13D mutations enhance basal Noxa levels by activating ERK2, thereby sensitizing premalignant epithelial cells to apoptosis upon exposure to cytotoxic agents [99]. This provides a mechanistic link between oncogenic mutations and apoptotic priming. However, findings in colorectal cancer cells indicate that Noxa levels do not consistently change with KRAS mutations, despite baseline levels being higher than those in premalignant epithelial cells [99]. This discrepancy highlights the context-dependent regulation of Noxa and suggests that KRAS mutations may influence Noxa expression differently depending on the specific cellular background and stage of tumorigenesis.

In prostate cancer, Noxa and Puma are crucial in recurrence, with elevated Noxa expression frequently observed and strongly associated with adverse clinical outcomes [100]. This suggests that Noxa could serve as a valuable prognostic marker for prostate cancer progression and biochemical recurrence [100]. The consistent association of high Noxa expression with poor prognosis in prostate cancer contrasts with its pro-apoptotic function and warrants further investigation into how elevated Noxa might contribute to aggressive disease, perhaps by indicating a high level of apoptotic priming that the tumor has overcome through other compensatory anti-apoptotic mechanisms. Understanding these complex regulatory loops is crucial for translating Noxa’s prognostic potential into effective therapeutic strategies.

### p53 upregulated modulator of apoptosis (Puma): A central player in cancer apoptosis

Puma, also known as Bcl-2 binding component 3, plays a central and crucial role in mediating both p53-dependent and -independent apoptosis. Its primary function is to transmit death signals directly to the mitochondria. Puma achieves this by directly binding to and potently counteracting all known anti-apoptotic Bcl-2 family members, including Bcl-2, Bcl-xL, and Mcl-1 [101]. This broad binding capability is a defining characteristic of Puma among the BH3-only proteins. By binding these anti-apoptotic proteins, Puma displaces and thereby relieves their inhibition of the pro-apoptotic Bak and Bax proteins. This disruption shifts the balance towards apoptosis, leading to MOMP, the release of pro-apoptotic factors (like cytochrome c), and the subsequent activation of caspases, ultimately leading to controlled cell death. Specifically, Puma interacts with the BH1, BH2, and BH3 domains of anti-apoptotic proteins like Bcl-2 and Bcl-xL on the mitochondrial membrane [101].

While other BH3-only proteins also bind to anti-apoptotic Bcl-2 family members, Puma’s unique contribution lies in its broad and potent binding affinity to all major anti-apoptotic proteins. This allows Puma to act as a crucial activator of Bax and Bak by directly neutralizing the entire spectrum of their inhibitors. This contrasts with some other BH3-only proteins (like Noxa) which may have more restricted binding specificities (e.g., primarily to Mcl-1), or others (like Bad) which may primarily antagonize Bcl-2 and Bcl-xL but not Mcl-1. Puma’s pan-inhibitory activity against anti-apoptotic proteins makes it a particularly potent inducer of apoptosis. Deficiency or inhibition of Puma has been shown to significantly reduce apoptosis, thereby increasing the risk of cancer development and contributing to therapeutic resistance [102]. This highlights Puma’s essential role as a tumor suppressor. Originally discovered in colon cancer cells [103], Puma’s critical role in apoptosis is essential for maintaining cellular homeostasis and preventing uncontrolled cell proliferation, underscoring its significance in cancer biology. A key area for future research is to understand how tumors acquire Puma deficiency or inhibit their activity, as this could reveal novel therapeutic targets.

## Regulation of the Bcl-2 family members by the UPS

### Pro-survival Bcl-2 Family Proteins

#### Bcl-2

The regulation of Bcl-2 degradation by the UPS plays a vital role in cell death pathways, especially apoptosis [12, 104] (Table 2**) (**Fig. 2). However, the exact molecular mechanisms controlling Bcl-2 degradation have been elusive until recently. The E3 ubiquitin ligase Parkin regulates Bcl-2 by mediating its mono-ubiquitination, thereby increasing Bcl-2 stability. This modification enhances Bcl-2’s interaction with Beclin 1, inhibiting autophagy and linking Parkin to the regulation of cell survival pathways [105]. This finding presents a counterintuitive mechanism where ubiquitination, typically a degradation signal, stabilizes Bcl-2, emphasizing the complexity of UPS regulation.

**Table 2:** E3 ligases regulating Bcl-2 family proteins

Bcl-2 Family Protein Classification	Protein	Regulatory E3 ligase	Modification	Effect	Upstream Regulators	Therapeutic Relevance	References
Anti-apoptotic proteins	Bcl-2	Parkin	Mono-ubiquitination	Increased stability	-	Autophagy inhibition	[105]
		XIAP	Ubiquitination	Degradation	-	Dual antagonism by ARTS	[108, 109, 200]
	Bcl-xL	RNF183	Polyubiquitination	Degradation	IRE1α-dependent	Promotes apoptosis	[110]
	Mcl-1	FBXW7	Phosphorylation, Ubiquitination	Degradation	PI3K/AKT pathway	Enhances apoptosis	[111–113]
		MARCH5	Ubiquitination	Degradation	Noxa promotion	-	[65, 145, 147]
		MULE	Polyubiquitination	Regulates apoptosis	-	Disruption inhibits ubiquitination	[114, 146]
		SCFβ-TrCP	Polyubiquitination	Degradation	-	GSK3-induced tumor suppression	[116]
		TRIM17	Polyubiquitination	Degradation	-	Initiates apoptosis	[115]
Pro-apoptotic proteins (Effectors)	Bak	MARCHF5	Prevents activation	-	-	Apoptosis regulation	[121]
		Parkin	-	-	-	Inhibits apoptosis	[123]
	Bax	IBRDC2	Regulates levels	Prevents spontaneous activation	p53-mediated apoptosis	Modulates Bax stability, prevents cell death	[124]
		Parkin	Ubiquitination	Limits mitochondrial levels	Non-lethal stress	Anti-apoptotic effects	[122, 123]
		TRIM17	Ubiquitination	Degradation	Controls stability	Antagonizes apoptosis, promotes cancer survival	[125]
	Bok	Gp78 (E3)	Ubiquitination	Degradation	-	Constitutively active, regulated by ERAD	[137]
		TRIM28	Binds to mRNA	mRNA Degradation	-	Low levels correlate with better outcomes	[139, 140]
Pro-apoptotic proteins (Initiators)	Bad	-	-	-	-	-	-
	Bid	ITCH	Ubiquitination	Degradation	EGF stimulation	Apoptosis regulation	[126]
	Bik	Cullin5	Ubiquitination	Degradation	ER stress	Enhances apoptosis.	[133]
	Bim	β-TrCP	Phosphorylation, Ubiquitination	Degradation	-	Regulates apoptosis	[128]
	Noxa	MARCH5	-	-	-	Proteasomal turnover	[65, 145–147, 186]
	Puma	FBXL20	Degradation	Degradation	-	High expression promotes chemoresistance	[149]

More recently, specific mechanisms for Bcl-2 degradation have been elucidated. Studies have shown that cisplatin, a chemotherapy drug, induced down-regulation of Bcl-2 through a process involving dephosphorylation and ubiquitination of the protein, marking it for recognition and degradation by the proteasome [106]. A significant recent breakthrough involves ARTS (apoptosis-related protein in the TGF-β signaling pathway), a tumor suppressor protein, identified as a key factor in this regulatory process [107]. ARTS functions by promoting proteasome-mediated degradation of Bcl-2 and thereby stimulating cell death. ARTS forms a complex with both X-linked inhibitor of apoptosis (XIAP) and Bcl-2 [107]. Importantly, XIAP acts as an E3 ubiquitin ligase in this complex, promoting the addition of ubiquitin molecules to Bcl-2 [108]. This ubiquitination marks Bcl-2 for proteasomal degradation, which then reduces Bcl-2 levels in cells and promotes apoptosis.

The critical role of XIAP’s E3 ligase activity for Bcl-2 degradation was further confirmed by experiments using XIAP mutants: cells overexpressing an XIAP variant lacking the RING domain which is essential for its ligase function failed to exhibit Bcl-2 degradation. A mutant version of ARTS deleted at its unique C-terminal part has been shown to not bind to XIAP leading to increased levels of Bcl-2 [109]. This effect was also observed in mouse embryonic fibroblasts that lacked XIAP’s RING domain, showing higher Bcl-2 levels and demonstrating that XIAP’s E3 ligase activity is required to control Bcl-2 stability. The ARTS protein not only brings XIAP and Bcl-2 into a close spatial arrangement but also facilitates the specific ubiquitination of lysine 17 (K17) on Bcl-2. Mass spectrometry analysis confirmed this, revealing that K17 is a critical acceptor site for ubiquitination, and mutation at this site (K17A) effectively prevents Bcl-2 from undergoing caspase-3 cleavage during apoptosis. This unique positioning by ARTS is essential in promoting Bcl-2 degradation and lowering the threshold for apoptosis, especially under conditions that signal for cell death. By forming a ternary complex with XIAP and Bcl-2, ARTS essentially serves as a scaffold or adaptor, bringing these two proteins together to facilitate ubiquitination. In addition, ARTS appears to act as a dual antagonist in the apoptosis pathway, targeting both XIAP and Bcl-2 to regulate cell survival. In the context of cancer therapy, this dual role holds promise because it suggests that therapies aimed at enhancing ARTS function or mimicking its activity could simultaneously downregulate XIAP and Bcl-2, two critical anti-apoptotic proteins [108]. Consequently, ARTS presents a valuable therapeutic target for drug development in cancer treatments that seek to overcome apoptotic resistance. The critical role of XIAP’s E3 ligase activity for Bcl-2 degradation.

#### Bcl-xL

The ER-localized E3 ligase RNF183, characterized as a membrane-spanning RING finger protein, is situated in the ER and demonstrates conventional E3 ligase activities [110]. This provides a clear mechanism for its interaction with Bcl-xL. Varied treatments inducing sustained ER stress led to a post-transcriptional increase in RNF183 protein levels in an IRE1α-dependent manner. RNF183 interacts with and polyubiquitinates Bcl-xL, marking it for degradation [110]. As a result, RNF183 assumes a pivotal role in executing programmed cell death following prolonged ER stress, plausibly by prompting apoptosis *via* Bcl-xL. Elevated levels of RNF183 enhance their binding to Bcl-xL, thereby catalyzing polyubiquitination and subsequent proteasomal degradation. The gradual decline in Bcl-xL levels ultimately activates the intrinsic apoptotic pathway [110]. This discovery of RNF183 as a specific E3 ligase for Bcl-xL provides a novel therapeutic avenue. Future research should focus on identifying upstream activators of RNF183 or developing small molecules that enhance its E3 ligase activity to promote Bcl-xL degradation in cancer cells.

#### Mcl-1

The stability of Mcl-1, a key anti-apoptotic protein, is tightly controlled by a complex interplay of E3 ubiquitin ligases and deubiquitinases (DUBs). One prominent E3 ligase involved is FBXW7 (F-box and WD repeat domain-containing protein 7), which acts as a ubiquitin ligase [111, 112]. FBXW7 specifically targets substrates that have been phosphorylated by GSK3β (glycogen synthase kinase 3 beta), a serine/threonine kinase. When GSK3β phosphorylates Mcl-1 at specific sites, it creates a recognition motif for FBXW7, promoting FBXW7-mediated Mcl-1 ubiquitination and subsequent proteasomal degradation [111].

This regulatory axis is further influenced by the PI3K/AKT signaling pathway. AKT, a kinase downstream of PI3K, typically phosphorylates and inhibits GSK3β. Therefore, high AKT activity leads to inhibited GSK3β, which in turn reduces Mcl-1 phosphorylation and subsequent degradation by FBXW7, thus promoting Mcl-1 stability and cell survival. Conversely, inhibition of AKT, for example by small molecule inhibitors like API-1, removes this inhibitory effect on GSK3β. This allows GSK3β to phosphorylate Mcl-1, leading to its FBXW7-mediated ubiquitination and proteasomal degradation, thereby promoting apoptosis [113]. This highlights a point of convergence for targeting Mcl-1 by modulating upstream signaling pathways. Conversely, the kinase ERK phosphorylates Mcl-1 at distinct sites, which typically leads to increased Mcl-1 stability, highlighting the complex and sometimes opposing regulatory inputs from different signaling pathways. Further studies are essential to fully elucidate the intricate interactions and downstream effects of these converging pathways on Mcl-1 stability, particularly in specific cancer contexts.

In addition to FBXW7, Mcl-1 stability can be governed by a series of other E3 ligases, including MULE (Mcl-1 ubiquitin ligase E3), TRIM17, and SCFβ-TrCP (Skp1-Cullin1-F-box protein β-TrCP) [114–116]. MULE possesses a specific domain that directly interacts with Mcl-1. When this interaction is disrupted, Mcl-1 ubiquitination is inhibited, leading to its stabilization rather than degradation [117]. This diversity of E3 ligases regulating Mcl-1 suggests redundancy in its control, which might pose challenges for therapeutic targeting of a single ligase. The interplay between Mcl-1 and the BH3-only protein Noxa is also critical for its stability. Noxa not only displaces pro-apoptotic proteins from Mcl-1 but can also influence Mcl-1 degradation. Studies suggest that Noxa induction leads to the downregulation of the deubiquitinase Usp9x, which normally stabilizes Mcl-1 by removing ubiquitin tags. The reduction of Usp9x activity then resulted in increased ubiquitination and subsequent proteasomal degradation of Mcl-1 [104]. This Noxa-Usp9x-Mcl-1 axis presents a therapeutic vulnerability. For instance, the chemotherapeutic agent pemetrexed has been shown to induce apoptosis by modulating this pathway, highlighting potential therapeutic strategies for overcoming resistance in cancer treatment [62].

The crucial role of the mitochondrial E3 ubiquitin-protein ligase MARCH5 in Mcl-1 stability has also been explored. MARCH5’s role in maintaining Mcl-1 stability through a Noxa-dependent mechanism means that while downregulating MARCH5 might initially seem beneficial, it can lead to complex compensatory effects on Mcl-1 levels [66]. This highlights the nuanced understanding required when targeting Mcl-1 stability *via* its ubiquitin ligases or DUBs. Given Mcl-1’s frequent overexpression and its role in chemoresistance, particularly in cancers reliant on Mcl-1 for survival, inhibitors directly targeting Mcl-1 are actively being developed [67]. These inhibitors aim to bypass the endogenous UPS-mediated stabilization mechanisms, thereby promoting Mcl-1 degradation and enhancing apoptosis. These insights into the intricate regulatory mechanisms involving Mcl-1 and its interplay with various signaling and ubiquitin pathways emphasize the significant potential for targeted therapies that can enhance the efficacy of BH3 mimetic drugs and overcome resistance, ultimately improving cancer treatment outcomes. A key challenge moving forward is to precisely map the specific E3 ligases and DUBs that are most relevant in different cancer types and to identify strategies to selectively modulate their activity to destabilize Mcl-1.

### Pro-apoptotic Bcl-family proteins

#### Bad

Bad promotes apoptosis primarily by binding to anti-apoptotic Bcl-2 at the mitochondria, inhibiting its function and promoting Bax activation [118]. However, the cellular context significantly influences Bad’s activity. Phosphorylation of both Bcl-2 and Bad by JNK1 and other kinases disrupts this interaction, restoring Bcl-2’s ability to inhibit apoptosis and providing a clear mechanism for cells to evade death signals [119]. This highlights a key regulatory point where upstream kinases can dictate the fate of the cell. Additionally, mono-ubiquitination of Bcl-2 may enhance its association with Bad, potentially facilitating apoptosis, which presents an intriguing counter-regulatory mechanism where ubiquitination might promote, rather than inhibit, a pro-apoptotic interaction [118]. Conversely, AKT-mediated phosphorylation of Bad inhibits its pro-apoptotic activity, rendering cells more resistant to death [118]. Despite these insights into Bad’s regulation, information on the specific E3 ubiquitin ligases responsible for Bad degradation is currently unavailable, representing a significant knowledge gap. Identifying these ligases could open new therapeutic avenues to enhance Bad’s pro-apoptotic function in cancer cells.

#### Bak

The regulation of Bak by the UPS is less understood than that of other Bcl-2 family members, particularly compared to anti-apoptotic proteins, leaving considerable room for future discovery. One key insight is that the pro-apoptotic BH3-only protein Noxa promotes Bak activation by displacing it from Mcl-1, which subsequently leads to Mcl-1 degradation *via* the proteasome [120]. This suggests that Bak binding may inhibit Mcl-1 ubiquitination, and its release could trigger conformational changes that facilitate Mcl-1 degradation, highlighting the broader role of BH3-only proteins in not only activating pro-apoptotic effectors but also influencing the stability of their anti-apoptotic counterparts.

More recently, the E3 ubiquitin ligase MARCHF5 has been implicated in Bak regulation by preventing its activation and promoting its association with pro-survival proteins Mcl-1 and Bcl-xL [121]. Crucially, loss of MARCHF5 alters Bak conformation, leading to resistance against BH3-mimetic drugs. This reveals a novel mechanism of apoptosis regulation where an E3 ligase acts to keep Bak inactive rather than promoting its degradation when active. This finding may have significant therapeutic implications, suggesting that targeting MARCHF5 could potentially sensitize cancer cells to pro-apoptotic therapies [121]. However, the precise ubiquitination events on Bak mediated by MARCHF5, and the detailed consequences of these modifications on Bak’s function and localization, still require further elucidation. This remains an important area for future research to fully understand and exploit this regulatory axis.

#### Bax

Bax is regulated by the E3 ligase of the UPS, which influences its mitochondrial localization and overall activity. Parkin, an E3 ligase, plays a significant role in limiting Bax’s mitochondrial levels under non-lethal stress conditions [122]. This is a crucial finding, as it suggests Parkin acts as an anti-apoptotic regulator by preventing Bax accumulation at mitochondria. Parkin has been shown to ubiquitinate endogenous Bax in primary neurons, cell culture models, and in vitro assays, directly influencing Bax activity and affecting cell survival [122, 123]. Thus, Bax is considered a key substrate mediating Parkin’s anti-apoptotic effects, as ubiquitination-resistant Bax retains apoptotic function, whereas lysine-mutant Bax fails to replicate Parkin’s protective role [122]. This directly implicates ubiquitination in modulating Bax’s apoptotic potential.

Another important regulator is IBRDC2, an IBR-type RING-finger E3 ubiquitin ligase, which targets Bax in p53-mediated apoptosis [124]. IBRDC2 regulates Bax levels and prevents its spontaneous activation and cell death, suggesting a role in maintaining cellular homeostasis by controlling Bax [124]. IBRDC2 interacts with active Bax, translocates to mitochondria upon apoptosis induction, and modulates Bax stability in a ubiquitination-dependent manner influenced by Bcl-xL expression [124]. This indicates a complex interplay between IBRDC2, Bax, and anti-apoptotic Bcl-xL. Additionally, TRIM17 has been identified as an E3 ligase that interacts with Bax and promotes its ubiquitination and proteasomal degradation, thus antagonizing apoptosis [125]. The discovery of multiple E3 ligases regulating Bax highlights the complexity and redundancy in its control, which could pose challenges for therapeutic strategies aiming to modulate Bax stability. Further research is needed to delineate the specific contexts and conditions under which each of these E3 ligases primarily regulate Bax, and how their coordinated or competitive actions impact the apoptotic threshold in cancer.

#### Bid

The BHl for apoptosis driven by death receptor signaling, typically functioning through its cleaved, active form, tBid. A key regulatory mechanism involves ITCH, an E3 ubiquitin ligase with a HECT domain, which specifically targets tBid for ubiquitination and subsequent degradation [126]. This suggests that ITCH functions in an anti-apoptotic role by removing the active form of Bid. This degradation pathway is notably activated by epidermal growth factor (EGF) stimulation, establishing a direct connection between EGF signaling (a common oncogenic pathway) and the regulation of apoptosis through tBid stability [126]. This offers a potential therapeutic vulnerability, where inhibiting ITCH might sensitize cancer cells to death receptor-induced apoptosis.

While Bid has a long lifespan, tBid has a relatively short half-life, primarily due to its breakdown by the 26 S proteasome. Interestingly, tBid interaction with Bax or Bcl-2 does not appear to affect this degradation [46]. Studies in human and mouse models show that cancer cells have high levels of 26 S proteasome, which may contribute to the breakdown of tBid, leading to unopposed anti-apoptotic Bcl-2 family protein functions [71]. This presents a critical insight into how cancer cells can disarm a potent pro-apoptotic signal.

Opposing Bcl-2, Bid regulates apoptosis in liver cells, with studies demonstrating that Bid-deficient mice exhibit resistance to Fas-induced hepatocellular apoptosis. Research continues to investigate Bid expression across conditions such as chronic hepatitis, liver cirrhosis, HCC, and liver metastases. In the absence of Bid, hepatocytes show resistance to Fas-induced apoptosis, highlighting Bid as a potential target for cancer prevention strategies aimed at enhancing apoptosis and reducing tumor survival [73, 127]. A significant gap remains in understanding the full spectrum of E3 ligases that target Bid, particularly in different cellular contexts and in response to diverse apoptotic stimuli.

#### Bim

Bim promotes cell death by facilitating Bax activation and maintains a balance with the anti-apoptotic protein Mcl-1. Bim’s stability is regulated through post-translational modifications. Specifically, ERK1/2 phosphorylation of Bim enhances its interaction with other proteins, notably increasing its susceptibility to degradation mediated by the E3 ubiquitin ligase β-TrCP [128]. This phosphorylation-dependent degradation of Bim is a crucial regulatory mechanism for controlling apoptosis and maintaining cellular homeostasis [104]. The inverse relationship between Bim levels and ERK activity is a well-established mechanism for tumor survival. Targeting the ERK/β-TrCP axis could offer a strategy to stabilize Bim and re-sensitize cancer cells to apoptosis. However, the precise mechanisms by which other kinases or phosphatases might influence Bim’s stability through alternative phosphorylation sites or protein-protein interactions remain to be fully elucidated.

#### Bik

Bik exhibits complex regulation by the UPS, often linked to its phosphorylation state, which contributes to its paradoxical roles in cancer. Bik interacts with phosphorylated Erk1/2 (p-Erk1/2) in the cytosol, leading to Bik sequestration and eventually its degradation [129, 130]. Specifically, within the Ras-Raf-Mek1/2-Erk1/2 pathway, Erk1/2 phosphorylates Bik at serine 124 (S124), triggering Bik ubiquitination and proteasomal degradation [131]. Experiments have shown that inhibiting p-Erk1/2 with the ERK1/2 inhibitor PD184352 disrupts Bik phosphorylation, indicating that p-Erk1/2 is the crucial kinase responsible for phosphorylating Bik at this site [131]. Phosphorylation at S124 is associated with decreased stability and a shortened half-life of Bik, contributing to poor prognosis in BC, especially triple-negative breast cancer (TNBC). This highlights a direct mechanism by which oncogenic signaling pathways can undermine Bik’s tumor-suppressive function.

In v-src-transformed cells, Bik is further targeted by Src tyrosine kinase, which promotes ubiquitination at lysine 33 (K33) and subsequent proteasomal degradation [132]. Mutating K33 to an arginine residue (K33R) stabilizes Bik, providing strong evidence for this degradation pathway. However, the specific E3 ligase involved in this Src-mediated degradation remains unidentified, representing a key knowledge gap. Interestingly, phosphorylation of Bik at distinct residues (T33 and S35) has an opposing effect, enhancing its pro-apoptotic function rather than promoting degradation, as seen with the BikDD mutant (a mutant where Bik’s T33 and S35 are phosphorylated to enhance apoptotic potential) [132]. This mutant, however, is also subject to ubiquitination and degradation *via* the same S124 phosphorylation pathway when treated with ER stress-inducing agents, highlighting the dual role of phosphorylation in modulating Bik stability and function.

A recent study identified ASB11, an ER-resident adaptor protein, as a key mediator in Bik degradation. Under ER stress, ASB11 is transcriptionally activated by the IRE1α effector XBP1s, which stimulates Cullin5 (Cul5)-mediated ubiquitination and subsequent proteasomal degradation of Bik [133]. This degradation pathway is part of a broader stress adaptation mechanism, as it regulates cell survival under adverse conditions. Unlike typical ER-associated degradation (ERAD) that removes damaged or misfolded proteins [134], this Cullin5-ASB11 pathway targets functional Bik, which suggests a regulatory role in controlling apoptosis rather than solely a quality-control function. Disrupting this degradation pathway by either inhibiting ASB11 or knocking down Cul5 stabilizes Bik, which has been shown to increase cell apoptosis, supporting a therapeutic angle for cancer treatments where enhanced Bik stability may be desirable [133]. Additionally, research has shown that the AAA + ATPase p97, or valosin-containing protein (VCP), is required for the degradation of ubiquitinated Bik [135]. p97 and its cofactor complex (UFD1L-NPL4) facilitate the proteasomal degradation of ubiquitinated Bik under ER stress, further underlining the complexity and specificity of the regulatory networks that govern Bik stability [135]. These diverse and sometimes contradictory regulatory mechanisms underscore the challenges in therapeutically targeting Bik, and future efforts should focus on deciphering the dominant degradation pathways in specific cancer subtypes.

#### Bok

Bok promotes KRAS-driven lung cancer progression in a p53-dependent manner. Similar to pro-apoptotic proteins Bak and Bax, Bok is located mainly in the ER, where its activity is regulated by ERAD of Bak [136, 137]. Bok initiates cell death in response to ER stress and aids in DNA damage response through the TP53 pathway. Studies show that Bok deficiency leads to increased cellular damage [138].

Beyond its pro-apoptotic functions, Bok also regulates apoptosis-independent functions, such as the unfolded protein response (UPR), cellular proliferation, metabolism, and autophagy. Bok levels are often low in cancers due to mechanisms like mRNA instability [139]. TRIM28, an overexpressed protein in cancers linked to poor prognosis, binds to regulatory elements in Bok mRNA, promoting its degradation [140]. This provides a clear mechanism for how cancer cells suppress Bok expression. Consistently, high Bok and low TRIM28 levels correlate with increased survival in cancers like HCC and kidney cancer [139], further supporting Bok’s role as a tumor suppressor despite its pro-tumorigenic role in KRAS-driven lung cancer. This discrepancy in its function across cancer types represents a significant area of disagreement in the literature and warrants further investigation.

Unlike Bak and Bax, which exist in inactive forms that require activation, Bok shows intrinsic instability [110] and a substantial amount of Bok proposedly is in its active form [137]. Thus, to avoid fundamental apoptosis initiation, Bok is broadly controlled by proteasomal degradation. The mitochondrial apoptotic pathway typically requires Bak and Bax, but Bok, can independently initiate MOMP [136, 141]. Unlike Bak and Bax, Bok is constitutively active and resistant to antiapoptotic Bcl-2 proteins, regulated instead by the ERAD pathway [136, 141]. Bok is specifically marked for proteasomal degradation *via* ubiquitination by the Gp78 E3 ligase complex and VCP/p97. If ERAD or proteasome activity is impaired, Bok stabilizes, inducing apoptosis without needing Bak or Bax [137]. This unique regulatory mechanism positions Bok as an “apoptosis sensor” that triggers cell death when protein degradation pathways are compromised [137, 141]. This highlights a promising therapeutic angle, where interfering with Bok’s degradation could be a strategy to induce apoptosis in tumors resistant to conventional therapies.

Bok’s high turnover is maintained by its interaction with ERAD and other Bcl-2 family proteins, with misfolded Bok proteins rapidly processed by the proteasome. This ERAD system is tightly linked to the UPR, which manages cellular stress and protein quality control. ERAD not only handles misfolded protein clearance but also stabilizes key UPR elements like IRE1a, maintaining ER homeostasis and folding capacity [142]. In heart disease, dysregulation of proteasomal or lysosomal degradation impairs protein control, contributing to pathologies like congestive heart failure, underscoring the importance of balanced protein turnover in cell survival and health [143]. Given the dual nature of Bok’s role in cancer (pro-tumorigenic vs. tumor suppressive depending on context) and its unique regulation, a significant knowledge gap lies in precisely identifying the conditions and molecular determinants that dictate its function, and how this can be exploited for targeted therapy.

#### Noxa

Degradation of the BH3-only Noxa protein in vivo occurs independently of ubiquitin, facilitated by the 19 S regulatory particle (RP) subunit of the 26 S proteasome. When 26 S proteasomes are disrupted, Noxa rapidly accumulates, suggesting a unique degradation pathway. Noxa’s unstructured BH3 domain may act as a degron, possibly interacting with an unidentified polyubiquitinated adaptor protein that targets Noxa for 19 S RP degradation starting from its unstructured region [144]. This “ubiquitin-independent” proteasomal degradation mechanism for Noxa is a significant area of ongoing research and distinguishes its regulation from other Bcl-2 family members. This is further supported by the observation that a lysine-less Noxa-LL mutant, resistant to ubiquitination, still relies on the intact 26 S proteasome for degradation [144].

Noxa plays a crucial role in regulating the stability of Mcl-1, an anti-apoptotic protein essential for cell survival. Noxa promotes Mcl-1 degradation, particularly through the mitochondrial E3 ligase MARCH5, which collaborates with UBE2K and MTCH2 in marking Mcl-1 for proteasomal breakdown [145]. This highlights a direct interaction between a pro-apoptotic and an anti-apoptotic protein that leads to the degradation of the latter. Furthermore, Noxa overexpression also disrupts the USP9X/Mcl-1 interaction, increasing polyubiquitinated Mcl-1 forms and its association with the E3 ligase Mule, further driving Mcl-1 degradation [146]. This complex interplay of Noxa with multiple Mcl-1 regulatory components underscores its potent ability to destabilize Mcl-1.

During mitotic arrest induced by microtubule-targeting agents (MTAs), MARCH5 regulates Mcl-1/Noxa levels, delaying apoptosis onset. The inhibition of MARCH5 amplifies Noxa accumulation, sensitizing cancer cells to MTAs by inducing apoptosis in mitotically arrested cells and cells that escape mitosis [147]. This provides a clear therapeutic strategy: targeting MARCH5 to enhance Noxa levels and improve chemotherapy efficacy. Cancer cells may also evade genotoxic chemotherapy by enhancing Noxa degradation, with the deubiquitylating enzyme UCH-L1 regulating Noxa stability by removing ubiquitin chains that would otherwise mark it for proteasomal degradation [148]. The existence of a deubiquitinase that stabilizes Noxa, despite its ubiquitin-independent degradation, adds another layer of complexity and suggests a potential compensatory mechanism in cancer cells. Unraveling the complete network of factors that regulate Noxa stability is crucial for maximizing its therapeutic potential as an Mcl-1 antagonist.

#### Puma

Puma and Bax are among the most well-known inducers of apoptosis and expression levels of Bax and Puma are controlled at the posttranslational level by phosphorylation [149]. However, the mechanistic regulation of these proapoptotic proteins remains largely unexplored. A switch between two signaling kinases AKT1 and GSK3α/β modulates the functional activity of these proapoptotic regulators, thereby determining cell survival or death [149]. This highlights how upstream signaling pathways directly influence the apoptotic machinery.

More specifically regarding the UPS, RNAi-mediated ablation of FBXL20, an F-box protein that is part of an E3 ligase complex, results in increased levels of Puma as well as Bax, which further enhances the sensitivity of cancer cells to chemotherapeutic drugs [149]. This suggests that FBXL20 normally promotes the degradation of Puma and Bax, thereby dampening apoptosis. The high-level expression of FBXL20 in cancer cells, which reduces therapeutic drug-induced apoptosis and promotes chemoresistance, identifies it as a critical target. Therefore, the importance of targeting FBXL20 in cancers in conjunction with chemotherapy may represent a promising anticancer strategy to overcome chemoresistance. However, despite this important finding, the complete identity of the E3 ubiquitin ligase complex (beyond just FBXL20) involved in the proteasomal degradation of Bax and Puma, and the precise ubiquitination sites, remained elusive at the time of this study [149]. Further research is clearly needed to fully characterize these regulatory mechanisms to develop more effective strategies to unleash Puma and Bax-mediated apoptosis in drug-resistant cancers.

## Therapeutic potential of targeting the Proteasome-Bcl-2 axis

### Proteasome inhibitors: modulating the Bcl-2 axis and synergistic therapeutic strategies

**Fig. 3:**
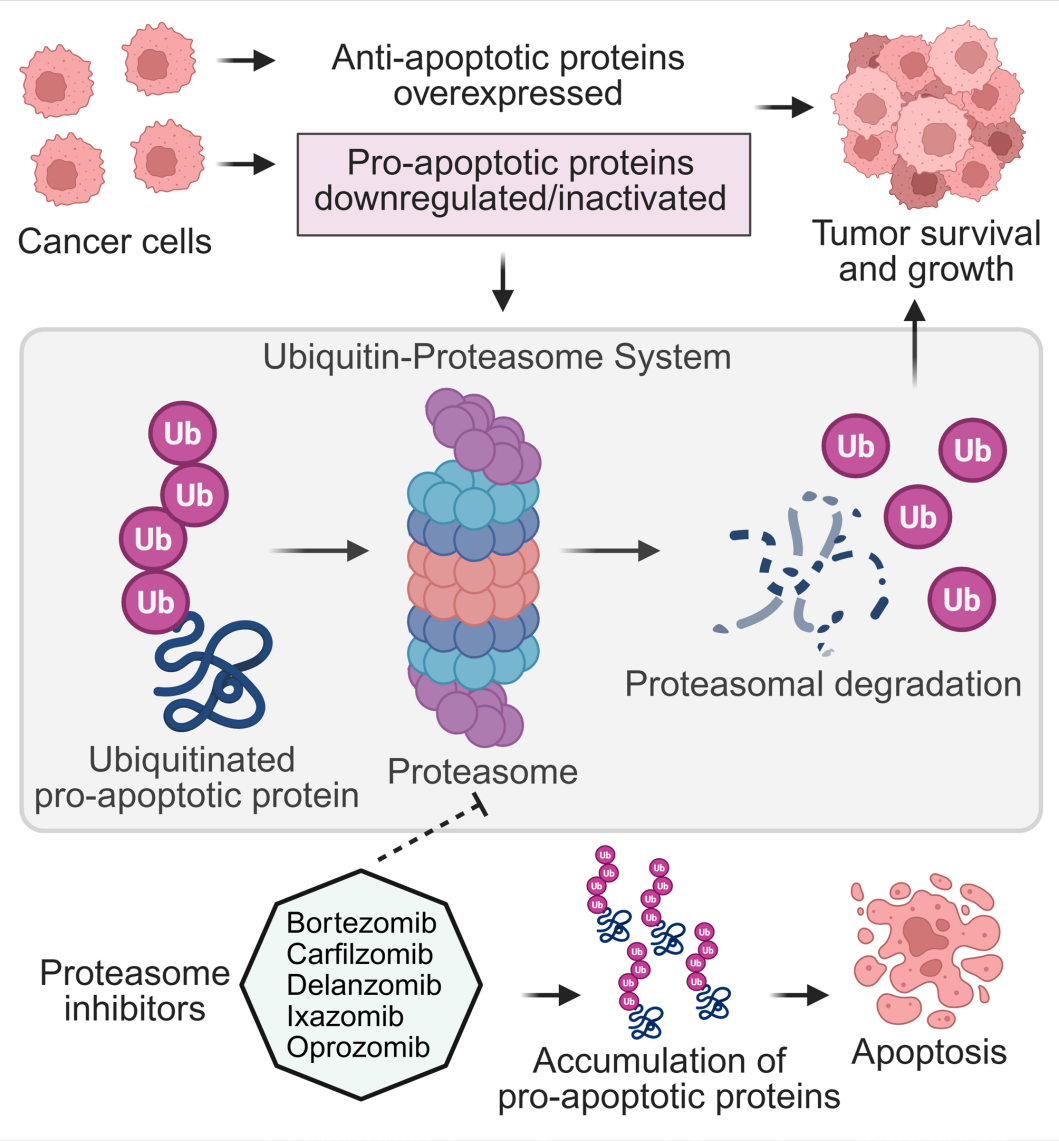
Proteasome inhibition restores apoptosis in cancer by preventing degradation of pro-apoptotic proteins. This figure illustrates how the ubiquitin-proteasome system (UPS) contributes to tumor cell survival by targeting pro-apoptotic proteins for degradation. In cancer cells, anti-apoptotic proteins are frequently overexpressed, while pro-apoptotic proteins are downregulated or degraded *via* the UPS, promoting tumor growth. **Top panel**: Cancer cells upregulate anti-apoptotic proteins and suppress pro-apoptotic proteins, enabling survival and uncontrolled proliferation. **Center panel**: Pro-apoptotic proteins are ubiquitinated and targeted for proteasomal degradation. The figure depicts the 26 S proteasome recognizing polyubiquitinated proteins and degrading them into peptides, thereby suppressing apoptosis. **Bottom panel**: FDA-approved proteasome inhibitors (Bortezomib, Carfilzomib, Delanzomib, Ixazomib, Oprozomib) block proteasomal activity, leading to accumulation of pro-apoptotic proteins and restoration of apoptotic signaling in tumor cells. By preventing the degradation of key pro-apoptotic factors, proteasome inhibitors re-activate apoptosis, offering a therapeutic strategy against proteasome-addicted tumors. Purple circles labeled “Ub” indicate ubiquitin. Dashed arrows show degradation; blocked lines indicate inhibition

Cancer cells exploit the UPS to degrade pro-apoptotic proteins [150, 151], enabling them evade apoptosis and promote tumor growth and progression [152] (Table 2). Targeting the UPS with proteasome inhibitors has emerged as a promising strategy for cancer therapy. Proteasome inhibitors disrupt the cellular protein degradation machinery, leading to the accumulation of unfolded proteins and the induction of apoptosis (Fig. 3). The UPS facilitates cancer cell survival by targeting pro-apoptotic proteins for proteasomal degradation, thereby inhibiting apoptosis. As illustrated in Fig. 3, this process is exploited in cancer to maintain anti-apoptotic dominance and enable tumor progression. Proteasome inhibitors such as Bortezomib, Carfilzomib and Ixazomib block this degradation, allowing pro-apoptotic proteins to accumulate and reinitiate apoptotic signaling, making them effective therapeutic agents in hematological malignancies and solid tumors (Table 3) [153, 154]. These drugs target the 20 S proteasome, inhibiting its proteolytic activity and disrupting various cellular processes. Originally, UPS-targeted therapies aimed at using proteasome inhibitors to combat cancers like multiple myeloma, resulting in several FDA-approved drugs for this purpose [153–156]. Bortezomib was the first proteasome inhibitor to be approved by the FDA in 2003. It inhibits the chymotrypsin-like activity of the 20 S proteasome, leading to the accumulation of pro-apoptotic proteins and the inhibition of NF-κB signaling [157, 158]. Carfilzomib is a more potent proteasome inhibitor than Bortezomib and is effective in patients who are resistant to Bortezomib [159]. However, it can cause significant side effects. Ixazomib is an oral proteasome inhibitor that is well-tolerated and has shown promising efficacy in clinical trials [159, 160], offering benefits for patients with refractory myeloma (Table 3) [161, 162].

**Table 3:** Clinical landscape of Bcl-2 and proteasome inhibitors in cancer therapy

Drug Name	Target	Cancer Type	Phase of Clinical Trial	Key Findings/Outcomes	References
Venetoclax	Bcl-2	Chronic Lymphocytic Leukemia (CLL), Small Lymphocytic Lymphoma	Chronic Lymphocytic Leukemia (CLL), Small Lymphocytic Lymphoma	Remarkable 79% overall response rate (ORR), with a 15-month progression-free survival rate of 69% in relapsed/refractory (R/R) CLL. Also effective in high-risk 17p-deleted R/R CLL (79% ORR).	[34]
Venetoclax	Bcl-2	Acute Myeloid Leukemia (AML)	Landmark VIALE-A trial	Monotherapy showed limited clinical activity (19% ORR) in patients unfit for intensive chemotherapy. Combination with hypomethylating agents (e.g., azacitidine) significantly enhanced remission rates and prolonged survival; nearly doubled overall survival (14.7 vs. 6 months) and led to a substantially higher complete remission (CR) rate.	[39]
Bortezomib	20 S Proteasome	Multiple Myeloma	FDA Approved (2003)	First proteasome inhibitor approved by FDA. Leads to accumulation of pro-apoptotic proteins and inhibition of NF-κB signaling.	[157]
Carfilzomib	Proteasome	Multiple Myeloma	Approved	More potent than Bortezomib. Effective in Bortezomib-resistant patients but can cause significant side effects	[203]
Ixazomib	Proteasome	Multiple Myeloma	Clinical trials, Approved	Oral proteasome inhibitor, well-tolerated, shown promising efficacy, offers benefits for patients with refractory myeloma.	[204]
Delanzomib	Proteasome	Multiple Myeloma	Phase II trials	Showed promising apoptotic effects, but Phase II trials were discontinued due to toxicity.	[205]
Oprozomib	Proteasome	Multiple Myeloma	Investigated in clinical trials, Under development	Showed promising apoptotic effects.	[206]

Additional proteasome inhibitors, such as Delanzomib and Oprozomib, have also been investigated in clinical trials. They have shown promising apoptotic effects in multiple myeloma, although Delanzomib’s toxicity led to its phase II trials being discontinued [163, 164]. Oprozomib and other novel proteasome inhibitors are currently under development. However, the long-term efficacy of proteasome inhibitors is often limited by the development of drug resistance [165, 166]. The efficacy of BH3 mimetic drugs like ABT-737 is largely contingent on Noxa expression; these drugs work by binding to Bcl-xL and Bcl-2, thereby allowing Bak to translocate to the mitochondria to promote apoptosis [167]. In terms of therapeutic applications, recent studies have revealed that combining BikDD with an IRE1α inhibitor, STF-083010, led to significant tumor suppression in TNBC models. Blocking the ASB11-dependent degradation of BikDD increases its stability, effectively sensitizing cancer cells to pro-apoptotic stimuli [133]. Further experiments demonstrate that ER stress induces ASB11 expression, which reduces Bik levels across multiple cell lines, whereas DNA damage counteracts this process by downregulating the IRE1α-XBP1s-ASB11 axis, thus stabilizing Bik [133]. This stability boost enhances the apoptotic response to DNA-damaging agents, such as doxorubicin. Bik stabilization by blocking ASB11-mediated degradation appears to amplify Bik’s apoptotic activity, suggesting that modulating this pathway could enhance responses to cancer treatments in TNBC and potentially in other cancer types. This combined treatment demonstrated a potent reduction in tumor growth, an effect not achieved with either treatment alone, underscoring the potential of ASB11 inhibition as an adjunct to Bik-based cancer therapies. Overall, targeting ASB11 and its associated degradation pathway may represent a novel and effective therapeutic approach for various cancers, including breast, colon, lung, and ovarian cancers, particularly in settings where traditional treatments are less effective due to drug resistance.

The mechanistic understanding of ARTS, XIAP, and Bcl-2 interactions provides a framework for potential therapeutic approaches. For instance, BH3-mimetic drugs that target Bcl-2 (such as ABT-199) could be used to disrupt the Bcl-2-ARTS interaction, freeing ARTS to interact with XIAP more effectively [108]. This could enhance Bcl-2 degradation and apoptosis, particularly in resistant cancer cells where XIAP and Bcl-2 are overexpressed. In summary, the interplay between ARTS, XIAP, and Bcl-2 in the UPS highlights an important regulatory axis in apoptosis, with the potential to be exploited in cancer therapy. By facilitating Bcl-2 degradation through targeted ubiquitination, ARTS reduces cellular resistance to apoptosis. This newly understood mechanism positions ARTS and its interaction with XIAP and Bcl-2 as promising targets in the development of next-generation therapies for cancers resistant to conventional treatments. Proteasome inhibitors were able to overcome Bcl-2-mediated protection from apoptosis. The proteasome activity in Bcl-2-over expressing cells accumulates the proapoptotic Bax protein to mitochondria cytoplasm, where it interacts with Bcl-2 protein. This event was followed by the release of mitochondrial cytochrome into the cytosol and activation of caspase-mediated apoptosis. In contrast, proteasome inhibition did not induce any apparent changes in Bcl-2 protein levels. In addition, treatment with a proteasome inhibitor increased levels of ubiquitinated forms of Bax protein, without any effects on Bax mRNA expression. Taken together, both in vivo and in vitro studies have demonstrated that Bax is regulated by an ATP- and ubiquitin-dependent, proteasome-mediated degradation pathway [168].

In cholangiocarcinoma patients, ABC294640 induces Noxa expression and Mcl-1 degradation, while Noxa knockdown prevents Mcl-1 degradation and apoptosis [93]. The combined action of Noxa and Bax triggers apoptosis, with Bortezomib enhancing Noxa expression by inhibiting proteasomal degradation. ABT-199 further boosts Noxa *via* the integrated stress response, with additional proteasome inhibition intensifying apoptosis and suggesting potential for combination therapies in cancer treatment [169].

Proteins tagged with ubiquitin are broken down by the proteasome, and cullin-RING ligases (CRLs) among the E3 ubiquitin ligases ubiquitinate key proteins essential for cell survival, promoting cancer cell proliferation [170]. CRL activation depends on Neddylation, which attaches the Nedd8 protein to targets, a process controlled by Nedd8-activating enzyme (NAE). The NAE inhibitor pevonedistat (MLN4924) has shown promise in this regard [171]. Beyond proteasome inhibition, targeting other components of the UPS, such as deubiquitinases, NEDD8-activating enzymes, and E3 ubiquitin ligases, offers additional therapeutic opportunities. For instance, pevonedistat is a NAE inhibitor that has shown promising results in clinical trials for acute myeloid leukemia [172]. Combinatorial therapies that combine proteasome inhibitors with other targeted therapies, such as HRS-4642 or sotorasib, may offer synergistic antitumor effects and overcome drug resistance [173].

In conclusion, proteasome inhibitors have revolutionized the treatment of multiple myeloma and other cancers. However, there is a still need for continued research to develop novel UPS-targeted therapies with enhanced efficacy and safety as well as addressing the persistent challenge of drug resistance.

### Harnessing combination therapies: unlocking the full potential of Bcl-2 Inhibition in cancer treatment

The advent of venetoclax, a selective Bcl-2 inhibitor, has reshaped the treatment landscape for cancers like chronic lymphocytic leukemia (CLL) and other lymphoid malignancies. As a standalone therapy, venetoclax has delivered impressive clinical responses. However, resistance often emerges over time, driven by changes in the apoptotic pathway machinery that allow cancer cells to evade programmed cell death [174]. To address this challenge, current strategies are shifting toward time-limited regimens and combination therapies, which aim to prolong efficacy and overcome resistance [175]. One promising avenue is the combination of venetoclax with epigenetic therapies, particularly histone deacetylase inhibitors (HDACis). These agents alter gene expression without modifying the DNA sequence and can re-sensitize cancer cells to apoptosis. This pairing has shown synergistic activity in both blood cancers and solid tumors [176, 177].

In AML, venetoclax is now commonly used in combination with hypomethylating agents like azacitidine [178] or decitabine [179], and even with low-dose cytarabine [180]. These regimens are particularly effective in older patients or those ineligible for intensive chemotherapy, offering high response rates with manageable side effects. In CLL, venetoclax combined with rituximab, a CD20-targeting monoclonal antibody, has become a standard of care for relapsed or refractory disease [181]. Meanwhile, pairing venetoclax with ibrutinib, a Bruton’s tyrosine kinase (BTK) inhibitor, offers a powerful dual-hit strategy, blocking survival signals while inducing apoptosis. This combination has proven effective in overcoming resistance and achieving deeper remissions [182]. Beyond chemotherapy and kinase inhibitors, venetoclax is being explored in immunotherapy combinations. By modifying the tumor microenvironment and promoting immune cell infiltration, Bcl-2 inhibition may enhance responses to immune checkpoint inhibitors, unlocking new potential for immuno-oncology strategies [183].

Another compelling combination involves proteasome inhibitors like bortezomib. These agents disrupt protein turnover, creating cellular stress that can be exploited when paired with venetoclax to trigger cancer cell apoptosis more effectively [169]. An especially promising multi-agent regimen includes venetoclax, ibrutinib, and obinutuzumab, which has shown high rates of minimal residual disease (MRD) negativity in CLL clinical trials, pointing to a future of potentially curative, fixed-duration therapy [184].

Taken together, these combination strategies underscore the importance of targeting multiple pathways simultaneously. By integrating Bcl-2 inhibitors with epigenetic drugs, immune modulators, kinase blockers, and proteasome inhibitors, researchers and clinicians are beginning to dismantle the complex survival networks that cancer cells depend on. Continued exploration of these synergistic interactions holds great promise for more personalized, potent, and durable cancer therapies.

### Clinical efficacy and limitations of proteasome inhibitors

Proteasome inhibitors have demonstrated significant clinical efficacy in the treatment of multiple myeloma and other hematological malignancies. By inhibiting the proteasome, these drugs disrupt the degradation of numerous proteins involved in cell cycle regulation, apoptosis, and inflammation [185] (Fig. 3). For instance, research reveals that the degradation of anti-apoptotic Mcl-1, mediated by Noxa during prolonged mitotic arrest (M-arrest), involves mitochondrial E3-ligase MARCH5, suggesting that inhibiting MARCH5 could enhance MTAs in cancers like HeLa and A549 [186]. Another promising molecule, BKA-073, is a Bak activator that selectively binds to Bak, promoting its oligomerization and apoptotic activity. In lung cancer models, BKA-073 has shown strong tumor suppression without significant toxicity and has reversed radiotherapy resistance in Bak-accumulated cells. Combined with the Bcl-2 inhibitor venetoclax, BKA-073 demonstrates synergistic effects, indicating the potential of Bak activators as a novel class of lung cancer therapies [187]. Key limitations of proteasome inhibitors include: (i) Drug resistance: Cancer cells can develop resistance to proteasome inhibitors through various mechanisms, such as upregulation of alternative proteolytic pathways, activation of survival signaling pathways, and increased expression of anti-apoptotic proteins. The UPS has become a focal point for developing new anti-cancer drugs, starting with proteasome inhibitors, which have shown efficacy in treating hematological malignancies [188]. However, resistance-both innate and acquired-limits their effectiveness [189]. Extensive research has identified pathways contributing to this resistance, allowing better patient selection for proteasome inhibitor therapies [190, 191]. Combination therapies are a promising strategy to overcome resistance and improve responses in cancers with limited sensitivity to proteasome inhibitors [188, 191]. Upregulation of certain E3 ligases, like cIAP, XIAP, and MDM2, often drives resistance, making these ligases prime targets for drug development, though challenges remain due to the diversity of E3 ligases and their multiple substrates [192]. (ii) Toxicity: Proteasome inhibitors can cause significant side effects, including peripheral neuropathy, thrombocytopenia, and fatigue [193, 194]. For example, Delanzomib showed promising apoptotic effects in multiple myeloma in clinical trials. However, its toxicity led to its phase II trials being discontinued [163, 164]. (iii) Limited efficacy in solid tumors: While proteasome inhibitors have shown promise in hematological malignancies, their efficacy in solid tumors has been more limited [195, 196].

### Emerging strategies to overcome resistance to proteasome inhibitors

Beyond small molecules, protein-targeting chimeras (PROTACs) have emerged as a novel approach, leveraging the UPS to degrade specific proteins and counteract resistance, though their clinical application faces design challenges [192]. UPS regulation is key to controlling apoptotic proteins, with 20 E3 ligases alone governing p53 levels, highlighting the system’s role in tumor suppression [197, 198]. Fewer E3 ligases regulate apoptosis inhibitors like Bcl-2 and XIAP, which may instead rely on protein-protein interactions for control [199–201]. Targeting protein degradation over inhibition can lower systemic drug concentrations, reducing side effects. This potential has spurred substantial efforts to harness UPS to promote apoptosis in cancer cells, with compounds targeting degradation showing promise for improving cancer treatment outcomes. While significant strides have been made in understanding the complex role of Bcl-2 family proteins in cancer, several challenges persist. Firstly, the intricate regulatory network of these proteins, often context-dependent, demands further elucidation for developing targeted therapies. Secondly, cancer cells frequently develop resistance to Bcl-2 inhibitors, necessitating the identification of resistance mechanisms and the development of strategies to overcome them. Thirdly, the potential for off-target effects and toxicity associated with targeting Bcl-2 family proteins underscores the need for highly specific inhibitors. Combining Bcl-2 inhibitors with other targeted therapies or chemotherapies may offer synergistic benefits and enhance therapeutic efficacy.

Future research should prioritize identifying novel targets within the Bcl-2 family and its regulatory pathways to develop more effective therapies. Exploring combinatorial approaches, such as combining Bcl-2 inhibitors with other targeted therapies or immunotherapies, may offer synergistic benefits and improve treatment outcomes. Understanding the mechanisms of drug resistance is crucial for developing strategies to overcome these challenges. Utilizing biomarkers to identify patients who are most likely to benefit from Bcl-2 targeted therapies can optimize treatment strategies. Continued preclinical and clinical studies are essential to validate the efficacy and safety of Bcl-2 inhibitors in various cancer types. By addressing these challenges and pursuing these future directions, we can harness the potential of Bcl-2 family proteins as therapeutic targets to improve cancer treatment outcomes.

## Conclusions

**Fig. 4:**
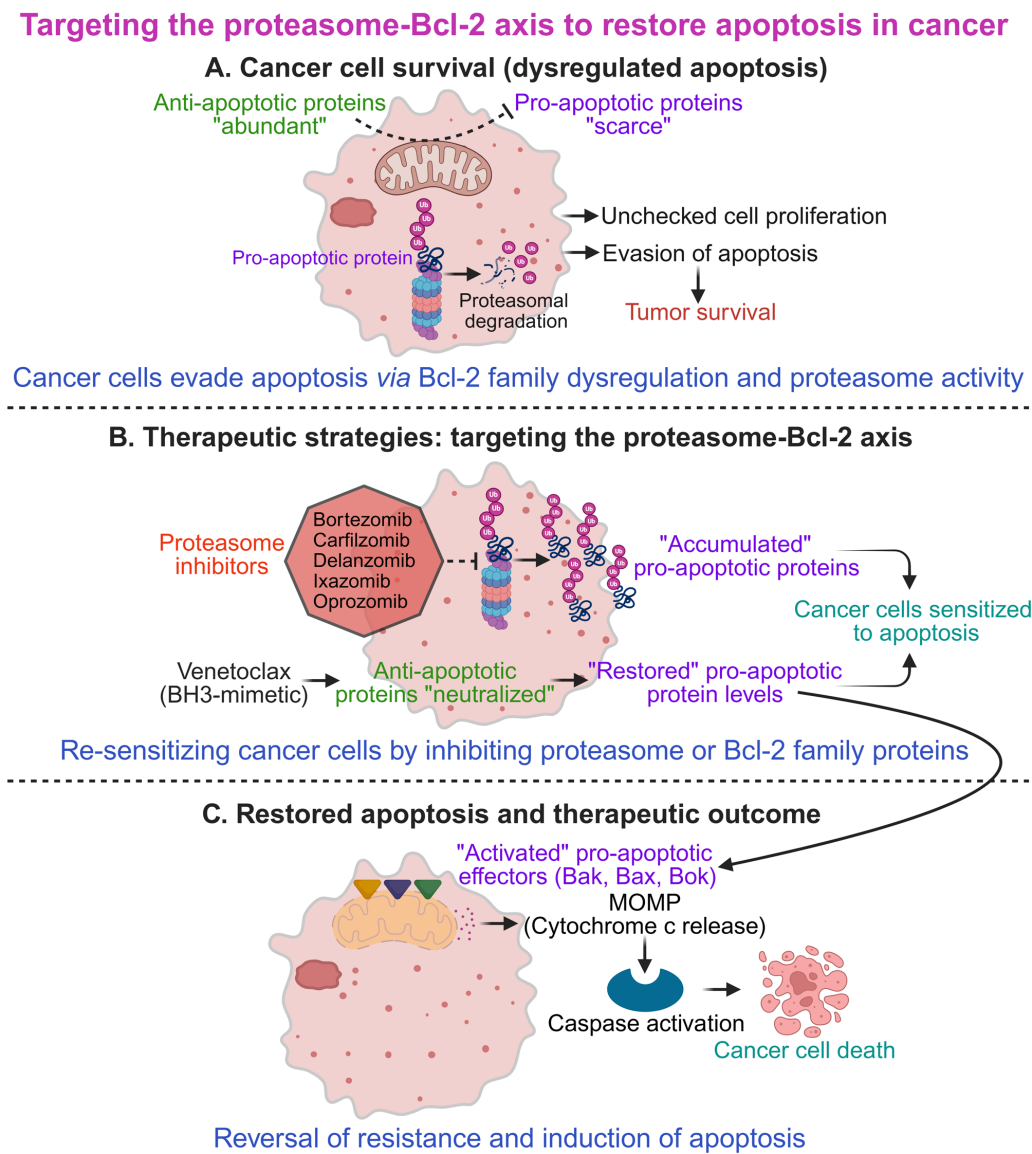
Targeting the proteasome-Bcl-2 axis to restore apoptosis in cancer. This summary illustration depicts the interplay between the ubiquitin-proteasome system (UPS), Bcl-2 family proteins, and therapeutic strategies aimed at reinstating apoptosis in cancer cells. (**A**) Cancer cell survival (dysregulated apoptosis): In cancer, apoptotic resistance is driven by overexpression of anti-apoptotic Bcl-2 family members (e.g., Bcl-2, Bcl-xL, Mcl-1, shown in green) and proteasomal degradation of pro-apoptotic proteins (e.g., Bax, Bak, Bok, and BH3-only proteins such as Bim, Noxa, Puma, etc. shown in purple). Active proteasomes degrade key pro-apoptotic regulators`, resulting in their reduced cellular levels and enabling evasion of mitochondrial outer membrane permeabilization (MOMP), caspase activation, and apoptosis. This imbalance promotes unchecked proliferation and tumor survival. (**B**) Therapeutic strategies: targeting the proteasome–Bcl-2 axis: The top branch illustrates proteasome inhibition using small molecules (e.g., Bortezomib, Carfilzomib, Delanzomib, Ixazomib, Oprozomib), which blocks degradation of pro-apoptotic proteins, leading to their accumulation and restored apoptotic potential. The bottom branch depicts BH3 mimetics (e.g., Venetoclax), which bind to and neutralize anti-apoptotic proteins, thereby releasing sequestered pro-apoptotic effectors. Both strategies restore the pro-apoptotic/anti-apoptotic balance, sensitizing cancer cells to apoptosis. (**C**) Restored apoptosis and therapeutic outcome: Rebalanced Bcl-2 signaling enables activation of pro-apoptotic effectors (Bak, Bax, Bok), MOMP, release of cytochrome c, and activation of the apoptosome and caspase cascade. This culminates in the execution of apoptosis, characterized by morphological changes such as membrane blebbing and nuclear fragmentation, ultimately leading to cancer cell death, tumor regression, and improved therapeutic outcomes. Color coding: Anti-apoptotic proteins (green), pro-apoptotic proteins (purple), proteasome inhibitors (red). Arrows indicate activation or consequence; dashed lines represent inhibitory effects

The intricate interplay between the ubiquitin-proteasome system (UPS) and the Bcl-2 family proteins represents a critical axis governing cellular apoptosis, a process frequently dysregulated in cancer. This review highlights how modulating the stability and function of Bcl-2 family members *via* proteasome-mediated degradation offers profound implications for cancer treatment. By precisely controlling the balance between pro- and anti-apoptotic proteins, the proteasome emerges as a pivotal regulator of cell fate and therapeutic response (Fig. 4).

Targeting this proteasome-Bcl-2 axis holds immense promise to usher in a new era of cancer therapy. The clinical success of proteasome inhibitors in hematological malignancies, coupled with the emerging efficacy of Bcl-2 family inhibitors (BH3 mimetics), underscores the therapeutic potential of this combined approach. Combining Bcl-2 inhibitors with proteasome inhibitors offers a synergistic strategy to destabilize cancer cell survival by restoring apoptotic signaling. This dual-targeted approach disrupts both protein homeostasis and anti-apoptotic defenses, paving the way for more durable and effective cancer therapies. By disrupting proteasome-mediated survival pathways or enhancing the degradation of anti-apoptotic Bcl-2 proteins, we can re-sensitize resistant tumors and restore apoptotic susceptibility. Figure 4 summarizes how therapeutic targeting of the proteasome-Bcl-2 axis can restore apoptosis in cancer cells, overcome resistance, and improve treatment outcomes.

However, challenges such as overcoming inherent or acquired drug resistance and mitigating toxicity remain. Future research must therefore focus on a deeper understanding of the specific E3 ligases and deubiquitinases that regulate individual Bcl-2 family members, identifying novel therapeutic vulnerabilities within this axis. Developing more potent and selective inhibitors and rigorously exploring rational combination therapies that synergistically target both the proteasome and Bcl-2 pathways, are crucial next steps. By strategically advancing our understanding and targeting of the proteasome-Bcl-2 axis, we can unlock transformative strategies, leading to more effective and durable treatments for a wide range of cancers.

## Data Availability

No datasets were generated or analysed during the current study.

## Abbreviations

5-FU: 5-fluorouracil
AML: Acute myeloid leukemia
ARSI: Androgen receptor signaling inhibitors
ARTS: Apoptosis-related protein in TGF-β signaling pathway
ASCL1: Achaete-scute homolog 1
Bad: Bcl-2-associated agonist of cell death
Bak: Bcl-2 homologous antagonist killer
Bax: Bcl-2-associated X protein
BC: Breast cancer
Bcl-2: B-cell lymphoma-2
Bcl-xL: B-cell lymphoma-extra large
BH: Bcl-2 homology domain
Bid: BH3-interacting domain death agonist
Bik: Bcl-2-interacting killer
Bim: Bcl-2 interacting mediator of cell death
Bok: Bcl-2-related ovarian killer
CLL: Chronic lymphocytic leukemia
CR: Complete remission
CRL: Cullin-RING ligases
CRPC: Castration-resistant prostate cancer
DSS: Disease-specific survival
DUBs: Deubiquitinases
E1: Ubiquitin-activating enzyme
E2: Ubiquitin-conjugating enzyme
E3: Ubiquitin ligase enzyme
EGF: Epidermal growth factor
EMT: Epithelial-mesenchymal transition
ER: Endoplasmic reticulum
ERAD: Endoplasmic reticulum-associated degradation
ERs: Estrogen receptors
ERα: Estrogen receptor alpha
ER+: Estrogen receptor-positive
FBXW7: F-box and WD repeat domain-containing protein 7
GC: Gastric cancer
GSK3β: Glycogen synthase kinase 3 beta
HCC: Hepatocellular carcinoma
Mcl-1: Myeloid cell leukemia-1
MOM: Mitochondrial outer membrane
MOMP: Mitochondrial membrane permeabilization
MTA: Microtubule-targeting agents
MULE: Mcl-1 ubiquitin ligase E3
NAE: Nedd8-activating enzyme
Noxa or PMAIP1: Phorbol-12-myristate-13-acetate-induced protein 1
NSCLC: Non-small cell lung cancer
ORR: Overall response rate
OS: Overall survival
PROTAC: Protein-targeting chimeras
Puma: p53 upregulated modulator of apoptosis
R/R: Relapsed/refractory
RP: Regulatory particle
SAC: Spindle assembly checkpoint
SCFβ-TrCP: Skp1-Cullin1-F-box protein β-TrCP
TNBC: Triple-negative breast cancer
UPR: Unfolded protein response
UPS: Ubiquitin-proteasome system
VCP: Valosin-containing protein
XIAP: X-linked inhibitor of apoptosis
